# Fungal inhibitory activity of sesquiterpenoids isolated from *Laggera pterodonta*

**DOI:** 10.3389/fpls.2025.1586984

**Published:** 2025-07-16

**Authors:** Yuxuan Liu, Jinliang Li, Guoxing Wu, Xiaoyun Wu, Yuhan Zhao, Xiao Ding, Xiaoping Qin

**Affiliations:** ^1^ State Key Laboratory for Conservation and Utilization of Bio-Resources in Yunnan, College of Plant Protection, Yunnan Agricultural University, Kunming, China; ^2^ Testing Center, Yunnan Dehong Institute of Tropical Agricultural Science, Ruili, China; ^3^ State Key Laboratory of Phytochemistry and Plant Resources in West China, Kunming Institute of Botany, Chinese Academy of Sciences, Kunming, China

**Keywords:** *Laggera pterodonta*, antibacterial activity, eudesmane-type sesquiterpenes, *Phytophthora nicotianae*, *Fusarium oxysporum*

## Abstract

The phytochemical diversity of plants represents a valuable reservoir for novel agrochemical agents. Our preliminary investigations identified pronounced antifungal efficacy in *Laggera pterodonta* extracts, although this species remains critically understudied. Herein, six eudesmane-type sesquiterpenes (1–6) were isolated from *L. pterodonta* and structurally characterized through spectroscopic analysis. Additionally, the antifungal activity of these compounds against six plant-pathogenic fungal species was evaluated: *Phytophthora nicotianae*, *Fusarium oxysporum*, *Alternaria alternata*, *Gloeosporium fructigenum* Berk, *Colletotrichum fructicola*, and *Botrytis cinerea*. The results showed that the six compounds exhibited varying degrees of inhibitory effects on the six plant pathogens. Compound 1 showed the strongest antifungal effect in a dose-dependent way, with half-maximal effective concentration (EC_50_) values of 12.56, 51.29, and 47.86 μg/mL against *P. nicotianae*, *F. oxysporum*, and *G. fructigenum* Berk, respectively. Compound 3 and compound 6 also showed notable inhibitory effects against *F. oxysporum* and *P. nicotianae* at 100 μg/mL, with inhibition rates of 58.82% and 73.92%, respectively. The minimum inhibitory concentrations (MICs) of compound 1 against *P. nicotianae* and *F. oxysporum* were 200 and 400 μg/mL, respectively. Mechanistic analysis revealed that compound 1 induced pronounced ultrastructural deformations in *P. nicotianae* and *F. oxysporum*, compromising membrane integrity and elevating permeability in both pathogens. Notably, the three bioactive compounds exhibited favorable ADMET (absorption, distribution, metabolism, excretion, and toxicity) profiles, demonstrating promising candidacy as novel herbicidal agents. These findings underscore their potential to advance phytogenic fungicide discovery.

## 1 Introduction

The advent of chemical pesticides revolutionized agricultural pest, disease, and weed management, substantially improving crop productivity. However, their prolonged use has precipitated the “3R” (resistance, residue, and resurgence) dilemma, along with pervasive environmental contamination, posing significant challenges to sustainable societal development. Consequently, plant-derived pesticides that are characterized by low mammalian toxicity, rapid degradability, high efficacy, and environmental compatibility have garnered increasing attention. The exploration and application of such eco-friendly agrochemicals now represent a priority research area in modern plant protection science (Yang et al., 2022; Prastiwi et al., 2023; Saravana, 2022). Numerous plant-derived active ingredients, including pyrethrins, picloram, neem, anethole, tea saponin, and nicotine, have been researched for their pesticidal potential, have been proven to be potentially bioactive against several pests, and have been used in agricultural applications. Moreover, their safety has been widely recognized and has been registered and commercialized successfully in several countries.

A growing interest in plant-derived fungicides has catalyzed the identification of approximately 1,400 plant species exhibiting antimicrobial (bacteriostatic and bactericidal) properties. These species biosynthesize a diverse array of antimicrobial phytochemicals, including terpenoids, alkaloids, flavonoids, phenolic acids, and other structurally distinct classes of secondary metabolites (Bhandari et al., 2021). For example, four terpenoids isolated from plants of the genus *Aromatica* showed good inhibitory effects on a variety of plant pathogens, and the inhibitory effects were selective and complementary (Ding et al., 2017). The phytochemical constituents of *Sophora flavescens* Aiton, including kurarinol and trifolirhizin (in addition to the well-characterized matrine), exhibit broad-spectrum inhibitory activity against various phytopathogenic bacteria (Wang et al., 2020). Similarly, *Salix myrtillacea* seed essential oil demonstrates potent fumigant efficacy against multiple plant pathogens (Liu et al., 2022). However, compared to the extensive exploration of plant-derived insecticides, phytogenic fungicides remain structurally homogeneous and inadequately studied, representing a promising yet underexplored frontier for agrochemical innovation (Zhang et al., 2023).

*Laggera pterodonta* (DC.) Benth., a member of the Asteraceae family (genus Laggera), has been employed in traditional Chinese medicine for centuries owing to its well-documented pharmacological properties, including antioxidant, antitumor, antimicrobial, and analgesic activities (Wang, 2019; Li et al., 2023; Xie et al., 2021). The chemical composition of *L. pterodonta* is dominated by flavonoids, eucalyptane-type sesquiterpenoids, and their derivatives, in addition to coumarins, triterpenoids, fatty acids, and volatile oil components (Lu et al., 2014; Wei et al., 2017; Chen et al., 2012).

Currently, extensive research has elucidated the diverse pharmacological activities of *L. pterodonta*. For instance, sesquiterpenes and flavonoids isolated from this species exhibit significant anti-inflammatory properties (Xie et al., 2021). Similarly, its phytochemical constituents, including chrysosplenetin and absinthin, demonstrate notable antioxidant activity (Li et al., 2013). Notably, the flavonoid 5,7,3′,4′-tetramethoxy-3-hydroxyflavone displayed potent antitumor efficacy *in vitro*, as validated by the 3-(4,5-dimethylthiazol-2-yl)-2,5-diphenyltetrazolium bromide (MTT) assay (Cao et al., 2011). In contrast, the agricultural applications of *L. pterodonta* remain underexplored. Preliminary studies have indicated that its ethanol extract exhibits contact toxicity against aphids (*Aphis gossypii*) and East Asian locusts (*Locusta migratoria manilensis*) (Gao et al., 2019), while petroleum ether extracts showed inhibitory effects against *Colletotrichum gloeosporioides*, the causative agent of oil tea anthracnose (Luo et al., 2022). Building on prior findings that the ethyl acetate extracts of *L. pterodonta* possess robust antimicrobial activity, we initiated a phytochemical investigation targeting eudesmane-type sesquiterpenes to identify novel antimicrobial agents.

**Figure 1:**
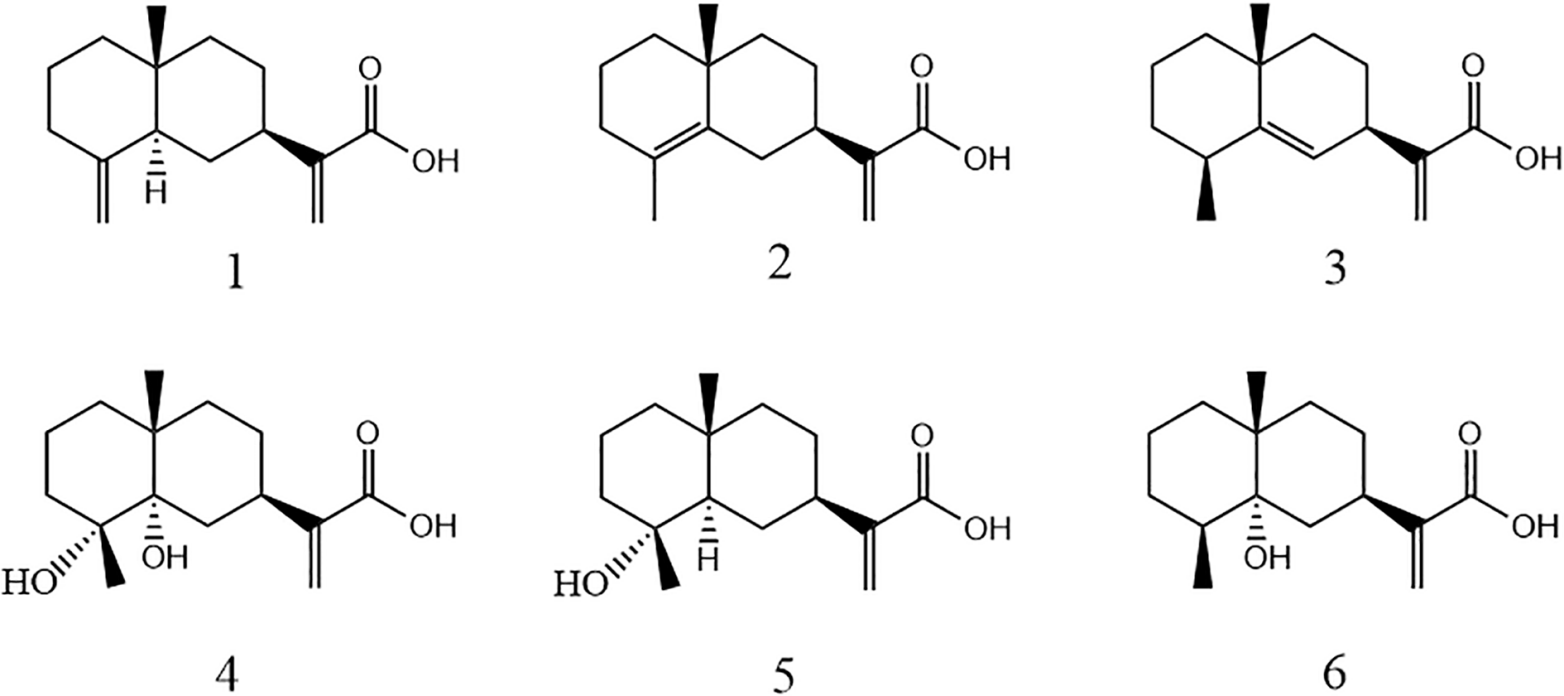
Chemical structures of the six compounds isolated from *Laggera pterodonta*.

In this study, six sesquiterpenoids were isolated from *L. pterodonta* and structurally characterized (**Figure 1**), with their antifungal efficacy evaluated against a panel of phytopathogenic fungi. To elucidate the mechanistic basis of their inhibitory activity, the most bioactive candidates were subjected to preliminary mechanistic investigation via scanning electron microscopy (SEM) and propidium iodide (PI) fluorescence staining assays. Furthermore, ADMET (absorption, distribution, metabolism, excretion, and toxicity) predictions for these compounds were conducted via the admetSAR platform to prioritize those candidates with the most potential to be developed as novel, plant-derived fungicides or as lead compounds for structure–activity optimization. This approach aims to establish both a phytochemical scaffold and an empirical foundation for advancing the discovery of next-generation agrochemicals.

## 2 Materials and methods

### 2.1 General experimental procedures

Silica gel (60–80, 200–300, and 300–400 mesh) and thin-layer chromatography (TLC) plates were purchased from Qingdao Ocean Chemical Co., Ltd. (Qingdao, China). MCI gel (75–150 μm) was obtained from Chengdu Scientific Biochemical Co. (Chengdu, China), and Sephadex LH-20 gel (40–70 μm) was sourced from Pharmacia (USA). Semi-preparative high-performance liquid chromatography (HPLC, Shimadzu, Kyoto Prefecture, Japan) was performed using ultrapure water (Milli-Q purification Systems, Millipore, Burlington, MA, USA) and HPLC-grade acetonitrile (J.T. Baker, Phillipsburg, NJ, USA), with a YMC-Pack ODS-A column (5 μm, 10 × 250 mm; Shenzhen Kemis Technology Co., Shenzhen, China). Reagent-grade solvents, including petroleum ether, ethanol, acetone, ethyl acetate, methanol (MeOH), and chloroform, were supplied by Qingdao Ocean Chemical Co., Ltd. (China).

NMR spectra were measured using Bruker 500 and 600 MHz spectrometers while using Tetramethylsilane (TMS) as the endogenous reference. Infrared spectra of KBr particles were measured using a BioRad FTS-135 spectrometer; rotameters were analyzed using a JASCO P-1020 digital polarimeter; and UV spectra were recorded using a Shimadzu UV-2401a. High-Resolution Electrospray Ionization Mass Spectrometry (HR-ESI-MS) was recorded on a triple quadrupole mass spectrometer (Agilent, USA).

### 2.2 Plant material

The above-ground parts of *L. pterodonta* were collected in July 2017 from the Baoshan countryside (25°C5′N, 99°C6′E), Yunnan Province, China. Prof. Hua Peng from the Kunming Institute of Botany, Chinese Academy of Sciences (CAS), identified the collected samples (via no. 1707016).

### 2.3 Extraction and isolation

Field-collected *L. pterodonta* (5 kg) was air-dried, mechanically pulverized, and exhaustively extracted with methanol (3 × 8 L) at ambient temperature. The combined filtrate was concentrated under reduced pressure to yield a crude extract (8 L), which was subsequently partitioned sequentially with petroleum ether and ethyl acetate (1:1, v/v; triplicate) to produce a petroleum ether-soluble fraction (68 g) and an ethyl acetate-soluble fraction (55 g). The ethyl acetate fraction was fractionated via silica gel column chromatography (10 × 100 cm) using a stepwise gradient elution of petroleum ether–acetone (100:1 to 1:1, v/v), yielding eight sub-fractions (Fr.1–Fr.8).

The Fr.4 (45.6 g) fraction was first purified using MCI column chromatography with a mobile phase of 10%–90% methanol–water in gradient elution to yield eight fractions (4A–4H). Fraction 4D was further eluted using forward silica gel column chromatography (mobile phase petroleum ether–ethyl acetate = 40:1–4:1) with gradient elution to yield five fractions (4D1–4D5). Fr.4D2 (3.9 g) was further purified using Sephadex LH-20 gel column chromatography (eluent was a mixture of methanol–dichloromethane = 6:4) and then again purified using semi-preparative HPLC [liquid phase conditions: the column was a YMC-pack ODS-A (5 mm, 10×250 mm), and the mobile phase was 58% acetonitrile in water at a flow rate of 3 mL/min, resulting in compounds 1 (100 mg, t_R_ = 59 min)), 2 (460 mg, t_R_ = 59 min), and 3 (135 mg, t_R_ = 55 min)]. Similarly, Fr.4F (2.1 g) was purified using Sephadex LH-20 gel column chromatography under methanol elution, followed by semi-preparative HPLC with a mobile phase of 47% acetonitrile in water, at a flow rate of 3 mL/min, to obtain compound 6 (1.9 g, t_R_ = 19 min).

Fr.5 (35.3 g) was purified using MCI column chromatography (gradient elution with 10%–90% methanol-water) to yield nine fractions (5A–5I). Fraction 5C was further purified using Sephadex LH-20 gel column chromatography (eluent was a mixture of methanol–dichloromethane = 6:4), followed by gradient elution using forward silica gel column chromatography (mobile phase petroleum ether–ethyl acetate = 40:1–1:1) to yield four fractions (5C1–5C4). After purifying Fr.5C2 (1.6 g) again using Sephadex LH-20 gel column chromatography (eluent was a mixture of methanol–dichloromethane = 6:4), it was purified by semi-preparative HPLC using 58% acetonitrile in water as the mobile phase at a flow rate of 3 mL/min to yield compounds 4 (67 mg, t_R_ = 27 min) and **5** (350 mg, t_R_ = 15 min).

### 2.4 Fungus material

*Phytophthora nicotianae*, *Fusarium oxysporum*, *Gibberella fructigenum* Berk, *Alternaria alternata*, *Colletotrichum fructicola*, and *Botrytis cinerea* strains were sourced from the Bio-Pesticide Development and Utilization Laboratory, Yunnan Agricultural University (Yunnan, China).

### 2.5 Screening of compounds for antifungal activity

The effect of the compounds on the mycelial growth of the pathogenic fungal strains was assessed using the mycelial growth rate method (Wang and Li, 2022). The compounds were dissolved in acetone to prepare 10 mg/mL stock solutions. Aseptically, 0.5 mL of each solution was added to 49.5 mL of sterile potato dextrose agar (PDA), yielding a drug-supplemented medium at a final concentration of 100 μg/mL. The medium was poured into Petri dishes, and 5-mm-diameter mycelial agar plugs (harvested from actively growing fungal colonies) were centrally inoculated onto each plate to standardize mycelial growth vigor. A solvent control containing an equivalent volume of acetone was included, and all treatments were performed in triplicate. The plates were then incubated at 27°C until the blank control filled the entire plate. Then, the diameter of the colonies was measured using the method of crossover, and the inhibition rate was calculated accordingly.


$$\text{Inhibition }\left(\%\right)=\left({\text{OD}}_{\text{CK}}-\text{ OD Compound}/{\text{OD}}_{\text{CK}}-\text{OD Fungus Cake}\right)\;\times \;100\%$$

When the compounds showed good inhibitory activity against the tested fungal strains, the master compound solution was diluted into five concentration gradients of compound solutions (10, 5, 2.5, 1.25, and 0.625 mg/mL). Then, the drug-containing medium was prepared at concentrations of 100, 50, 25, 12.5, and 6.25 μg/mL. The rest of the operation was performed as described above. When the blank control grew all over the plate, the colony diameter was measured, and the inhibition rate was calculated using the crossover method. The experimental data were processed and statistically analyzed using Microsoft Excel 2011 and SPSS 20.0 software, with the inhibition rate converted to the corresponding chance value to obtain the virulence regression equation and to find the half-maximal effective concentration (EC_50_) value.

### 2.6 Determination of MIC of compounds against *P. nicotianae* and *F. oxysporum*

Based on preliminary antifungal activity screening, compound 1—exhibiting broad-spectrum antifungal efficacy—was selected for further analysis. Its minimum inhibitory concentration (MIC) against *P. nicotianae* and *F. oxysporum* was assessed via the 96-well microdilution broth method (Isela et al., 2015). Prior to experimentation, *F. oxysporum* was maintained on PDA under standard conditions for a 7-day incubation period. Later, the spores were rinsed with sterile water and filtered through sterile gauze to remove the mycelium. The spores were then counted using a hemacytometer, and a spore suspension was prepared with a concentration of 1 × 10^6^ CFU/mL. *P. nicotianae* was first treated with 0.1% potassium nitrate to induce spore production, and then spores were counted as described above to make a spore suspension with a concentration of 1 × 10^6^ CFU/mL. Under aseptic conditions, 100 μL of a mixture of compounds and PDA and 100 μL of freshly prepared spore suspension (10^6^ spores/mL) were taken from a sterile 96-well plate and shaken gently to mix well so that the effective concentrations of the compounds in each column of the treatment group were maintained at 12.5, 25, 50, 100, 200, 400, and 800 μg/mL. At the same time, the culture medium was set as a blank control, and three replicates were set for each treatment. The plates were capped, sealed, and incubated at a constant temperature of 28°C. The MIC was designated as the lowest drug concentration achieving complete inhibition of fungal growth, corresponding to an absence of visible fungal proliferation in test wells relative to the untreated control.

### 2.7 Effect of compounds on mycelial morphology

The effect of compound 1 on the mycelial morphology of *P. nicotianae* and *F. oxysporum* was first observed using the coating plate method. A spore suspension of 50 μL was aspirated and coated on the plates containing the drug medium (the concentration of the compound was the EC_50_ for inhibiting fungal growth), and a plate without the compound was used as the control group, which was incubated in an incubator with a constant temperature and humidity of 28°C, and then observed under a microscope, photographed, and recorded in a timely manner for data collection.

SEM was employed to assess ultrastructural alterations in fungal morphology. *P. nicotianae* and *F. oxysporum* were pre-cultured on PDA, and 5-mm mycelial plugs were excised from colony margins. For treatment groups, plugs were transferred to a drug-supplemented medium, while a drug-free medium served as the control. Upon full mycelial colonization in the control group (CK), samples from both groups were sectioned, mounted on stubs, and flash-frozen in super-cooled liquid nitrogen (−196°C) for 2 min. Specimens were subsequently sublimated in a preparation chamber at −140°C for 15 min, followed by dual gold sputter coating (60 s each). Morphological analysis was conducted using a cryo-scanning electron microscope (Sigma 300, Zeiss, Jena, Germany) under high-vacuum conditions.

### 2.8 Cell membrane integrity assay

PI is unable to penetrate intact living cell membranes, but it can enter the cells through damaged cell membranes and bind to DNA to produce fluorescence. Therefore, the extent of damage to cell membranes by compounds was further evaluated in *P. nicotianae* and *F. oxysporum* after treatment with PI-stained compounds. Following the method of Bo et al. (2014), the drug-treated fungus was washed twice with Phosphate Buffered Saline (PBS) solution and gently transferred to a slide. Then, 50 μL of PI dye (1 g/L) was added dropwise, and stained in the dark for 15 min. Excess dye was washed away with PBS, covered with a coverslip, and observed under an inverted fluorescence microscope with 535 nm as the excitation wavelength and 617 nm as the emission wavelength.

### 2.9 ADMET prediction of active compounds

The Simplified Molecular Input Line Entry System (SMILES) notations of the bioactive compounds were submitted to the ADMET prediction module of the admetSAR platform (http://lmmd.ecust.edu.cn/admetsar1), with pharmacokinetic profiles generated using an automated algorithm execution.

## 3 Results and discussion

### 3.1 Structural identification of compounds

#### 3.1.1 Compound 1

Costic acid, colorless oil; the ^13^C-NMR and DEPT of the compound showed 15 carbon signals as one methyl, eight methylene, two hypomethyl, and four quaternary carbons; the molecular formula of the compound was determined to be C_15_H_22_O_2_ by combining with ESI-MS; the molecular weight of the compound was 234 (m/z: 233 [M − H]^−^). The NMR data were as follows: ^1^H-NMR (500 MHz, CDCl_3_) d: 2.3 (1H, m, H-1), 2.01 (1H, m, H-1), 1.58 (1H, m, H-2), 1.52 (1H, m, H-2), 1.59 (1H, m, H-3), 1.34 (1H, m, H-3), 1.89 (1H, d, *J* = 11. 3 Hz, H-5), 1.66 (1H, m, H-6), 1.22 (1H, m, H-6), 2.53 (1H, m, H-7), 1.61 (1H, m, H-8), 1.46 (1H, m, H-8), 1.59 (1H, m, H-9), 1.28 (1H, m, H-9), 6.31 (1H, s, H-13), 5. 68 (1H, s, H-13), 0.55 (3H, s, H-14), 4.40 (1H, d, *J* = 1.5 Hz, H-15), and 4.70 (1H, d, *J* = 1.5 Hz, H-15). ^13^C-NMR (150 MHz, CDCl_3_) d: 36.8 (C-1), 23.4 (C-2), 41. 8 (C-3), 150.7 (C-4), 49.8 (C-5), 29.9 (C-6), 39.3 (C-7), 27.3 (C-8), 41.0 (C-9), 36.8 (C-10), 145.3 (C-11), 172.5 (C-12), 124.8 (C-13), 16.4 (C-14), and 105.5 (C-15). The data were in general agreement with those of the reference (Kalliopi et al., 2017), which determined the structure of the compound.

#### 3.1.2 Compound 2

Isocostic acid, colorless oil, with the molecular formula C_15_H_22_O_2_ and a molecular weight of 234 (m/z: 233 [M − H]^−^) as determined by the ^13^C-NMR and ESI-MS of the compound. The NMR data were as follows: ^1^H-NMR (500 MHz, CD_3_OD) d: 6.15 (1H, d, *J* = 1.1 Hz, H-13), 5.59 (1H, s, H-13), 1.1 (3H, s, H-15), and 1.62 (3H, s, H-14). ^13^C-NMR (500 MHz, CD_3_OD) δ: 41.4 (C-1), 20.1 (C-2), 34.1 (C-3), 126.0 (C-4), 135.6 (C-5), 32.5 (C-6), 41.9 (C-7), 29.0 (C-8), 43.4 (C-9), 35.5 (C-10), 147.6 (C-11), 170.64 (C-12), 122.8 (C-13), 19.4 (C-14), and 25.0 (C-15). The NMR spectral data of the compound were in general agreement with literature comparisons (Cruz and Martinez, 1982), and the structure of the compound was determined.

#### 3.1.3 Compound 3

Eudesma-5,12-dien-13-oic acid, a colorless oily substance with the molecular formula C_15_H_22_O_2_ and a molecular weight of 234 (m/z: 233 [M − H]^−^) as determined by the ^13^C-NMR and ESI-MS of the compound. The NMR data were as follows: ^1^H-NMR (500 MHz, CD_3_OD) d: 1.21 (1H, m, H-1), 1.52 (1H, m, H-1), 1.85 (1H, m, H-2), 1.42 (1H, m, H-2), 1.55 (1H, m, H-3), 1.57 (1H, m, H-3), 2. 45 (1H, m, H-4), 5.19 (1H, s, H-6), 3.30 (1H, m, H-7), 1.92 (1H, m, H-8), 1.42 (1H, m, H-8), 1.44 (2H, m, H-9), 6.10 (1H, m, H-12), 5.52 (1H, m, H-12), 1.17 (3H, s, H-14), and 1.19 (3H, d, *J* = 7.6 Hz H-15). ^13^C-NMR (125 MHz, CD_3_OD) d: 43.15 (C-1), 18.54 (C-2), 34.4 (C-3), 39.67 (C-4), 149.63 (C-5), 124. 78 (C-6), 39.89 (C-7), 27.76 (C-8), 42.87 (C-9), 35.52 (C-10), 148.1 (C-11), 170.95 (C-12), 123.20 (C-13), 27.81 (C-14), and 23.57 (C-15). The NMR spectral data of the compound were in general agreement with literature comparisons (Ying et al., 2006), so the structure of the compound was determined.

#### 3.1.4 Compound 4

4α,5α-Dihydroxyeudesma-11(13)-en-12-oic acid, white acicular crystals. The molecular formula of the compound was determined to be C_15_H_24_O_4_, and its molecular weight was 268 (m/z: 267 [M − H]^−^) by ^13^C-NMR and ESI-MS. The NMR spectral data were as follows: ^1^H-NMR (500 MHz, CDCl_3_) d: 1.86 (1H, m, H-1), 1.43 (1H, m, H-1), 1.90 (1H, m, H-2), 1.84 (1H, m, H-2), 1.88 (1H, m, H-3), 1.40 (1H, m, H-3), 1.62 (1H, m H-6), 1.61 (1H, m, H-6), 2.95 (1H, m, H-7), 1.02 (1H, m, H-8), 1.70 (1H, m, H-8), 1.10 (1H, m, H-9), 0.1.02 (1H, m, H-9), 5.71 (1H, bs, H-13), 6.31 (1H, s, H-13), 1.20 (3H, s, H-14), and 1.28 (3H, s, H-15).^13^C-NMR (125 MHz, CDCl_3_) d: 17.52 (C-1), 29.70 (C-2), 36.32 (C-3), 75.16 (C-4), 76.06 (C-5), 26.20 (C-6), 34.41 (C-), 31.25 (C-7), 31.25 (C-5), 26.20 (C-6), 34.41 (C-), 31.25 (C-7), 31.25 (C-7), 31.25 (C-), 31.25 (C-) 7), 31.25 (C-8), 37.69 (C-9), 36.83 (C-10), 144.80 (C-11), 171.53 (C-12), 125.28 (C-13), 25.88 (C-14), and 22.20 (C-15). The NMR spectral data of the compounds were in general agreement with literature comparisons (Ying et al., 2006), and the structure of the compound was determined.

#### 3.1.5 Compound 5

Ilicic acid, white massive crystals; the ^13^C-NMR and DEPT combined with ESI-MS of the compound determined its molecular formula to be C_15_H_24_O_3_ and its molecular weight to be 252 (m/z: 253 [M + H]^+^). The NMR spectral data were as follows: ^1^H NMR (500 MHz, CDCl_3_) d: 0.89 (3H, s, H-14), 1.11 (3H, s, H-15), 1.25 (1H, m, H-6), 1.31~1.66 (2H, m, H-1), 1.31~1.66 (2H, m, H-8), 1.31~1.66 (2H, m, H-9), 1.31~1.66 (2H, m, H-8), 1.31~1.66 (2H m, H-9), 1.31~1.66 (2H, m, H-2), 1.81 (1H, m, H-3), 1.50 (1H, m, H-5), 1.89 (1H, d, *J* = 12.0 Hz, H-6), 2.50 (1H, m, H-7), 6.25 (1H, s, H-12), and 5.62 (1H, s, H-12). ^13^C-NMR (125 MHz, CDCl_3_) δ: 18.8 (C-15), 20.1 (C-2), 22.4 (C-14), 26.8 (C-8), 27.0 (C-6), 34.7 (C-10), 40.0 (C-7), 40.9 (C-9), 43.3 (C-1), 44.5 (C-3), 72.6 (C-4), 55.0 (C-5), 124.2 (C-12), 145.3 (C-11), and 171.6 (C-13). The NMR spectral data of the compound were in almost perfect agreement with literature comparisons (San et al., 1990), and hence, the structure was determined to be ilicic acid.

#### 3.1.6 Compound 6

5α-Hydroxy-4,15-dihydrocostic acid, white acicular crystals; the ^13^C-NMR and DEPT of the compound showed that the compound had 15 carbon signals of two methyls, two methylene groups, two hypomethyls, and four quaternary carbons, which was determined to have a molecular formula of C_15_H_24_O_3_ by combining with ESI-MS and a molecular weight of 252 (m/z: 234 [M − H20]^−^). The NMR spectral data were as follows: ^1^H-NMR (500 MHz, CDCl_3_) d: 6.24 (1H, s, H-13), 5.63 (1H, s, H-13), 0.90 (3H, s, H-15), 1.08 (3H, s, H-14), 2.05 (1H, ddd, *J* = 12.0, 12.0, 5.0 Hz, H-3), 1.34 (1H, d, *J* = 12.0 Hz, H-3), 1.58 (1H, dq, *J* = 7.0, 5.0 Hz, H-4), 1.20 (1H, dd, *J* = 12.5, 4.0 Hz, H-6), 1.86 (1H, dd, *J* = 12.5, 12.5 Hz, H-6), 3.06 (1H, m, H-7), 1.63 (1H, m, H-8), 1.53 (1H, m, H-8), 1.57 (1H, m, H-9), and 1.50 (1H, m, H-9). ^13^C-NMR (125 MHz, CDCl_3_) δ: 38.2 (C-1), 26.2 (C-2), 34.7 (C-3), 41.1 (C-4), 75.9 (C-5), 28.0 (C-6), 34.7 (C-7), 17.0 (C-8), 37.9 (C-9), 36.7 (C-10), 145.8 (C-11), 172.5 (C-12), 124.3 (C-13), 16.7 (C-14), and 21.8 (C-15). The data were in general agreement with those of the reference (Xie et al., 2011), which determined the structure of the compound.

### 3.2 Screening of compounds for fungal inhibitory activity

The antifungal activity of the six compounds was evaluated using a fungal growth rate assay (**Table 1**). At a concentration of 100 μg/mL, all compounds exhibited varying inhibitory effects against the tested phytopathogenic fungi. Notably, the eudesmane-type sesquiterpene acid compound 1 demonstrated pronounced inhibitory efficacy against *P. nicotianae*, *F. oxysporum*, and *Gibberella fujikuroi* Berk, with inhibition rates of 94.64%, 72.55%, and 70.46%, respectively. In contrast, its activity against *A. alternata*, *B. cinerea*, and *C. fructicola* was marginal, yielding inhibition rates of 15%–36%.

**Table 1:** Inhibition rates (%) of the compounds against six fungal pathogens.

Compound	*Phytophthora nicotianae*	*Fusarium oxysporum*	*Alternaria. alternata*	*Gloeosporium fructigenum* Berk	*Colletotrichum fructicola*	*Botrytis cinerea*
1	94.64 ± 1.39	72.55 ± 1.96	34.77 ± 2.16	70.46 ± 0.69	15.69 ± 1.96	35.29 ± 1.96
2	32.58 ± 1.63	16.34 ± 1.13	12.09 ± 1.13	16.67 ± 0.98	15.69 ± 1.96	15.03 ± 1.13
4	33.53 ± 0.34	58.82 ± 1.96	16.60 ± 0.93	25.16 ± 2.47	17.65 ± 0.59	18.56 ± 0.82
4	38.56 ± 2.26	13.86 ± 0.30	16.34 ± 1.13	13.86 ± 0.63	18.56 ± 2.46	8.69 ± 0.74
5	34.90 ± 1.03	17.78 ± 1.94	16.41 ± 1.67	19.54 ± 0.96	31.90 ± 1.24	13.95 ± 1.53
6	73.92 ± 0.86	15.82 ± 1.25	17.65 ± 1.96	32.09 ± 1.28	11.76 ± 1.69	13.07 ± 1.13

Compound 3 suppressed *F. oxysporum* mycelial growth by 58.82% but showed limited efficacy against the other five strains (16%–34% inhibition). Similarly, compound 6 inhibited *P. nicotianae* growth by 73.92%, while its activity against the remaining fungi ranged from 10% to 33%. The remaining three compounds (2, 4, and 5) displayed negligible antifungal effects against all tested pathogens, with inhibition rates consistently below 40%.

Structurally, compounds 1, 2, and 3 are isomers of each other, but compound 1 exhibited a broader spectrum and more fungal inhibitory activity as compared to the others. The differences among the three are manifested in the different positions of the double bonds; therefore, we speculate that the changes in the positions of the double bonds have a greater influence on the fungal inhibitory activity of eucalyptus-type sesquiterpene acid compounds and that the terminal double bonds of the compounds should exhibit greater activity. Meanwhile, compounds 5 and 6 are isomers of each other, and the structural difference between them is only the hydroxyl linkage site, but the inhibitory effect on *P. nicotianae* showed a significant difference. Combined with the structure and fungal inhibitory activity of compound 4, it is evident that the attachment of hydroxyl groups to C-3 of eucalyptane-type sesquiterpenoids significantly reduces their inhibitory effect against *P. nicotianae*. However, further studies are warranted to elucidate the broad-spectrum efficacy of this inhibitory effect against various phytopathogens.

### 3.3 Determination of the EC_50_ of compound 1 against three plant pathogenic fungi

**Figure 2:**
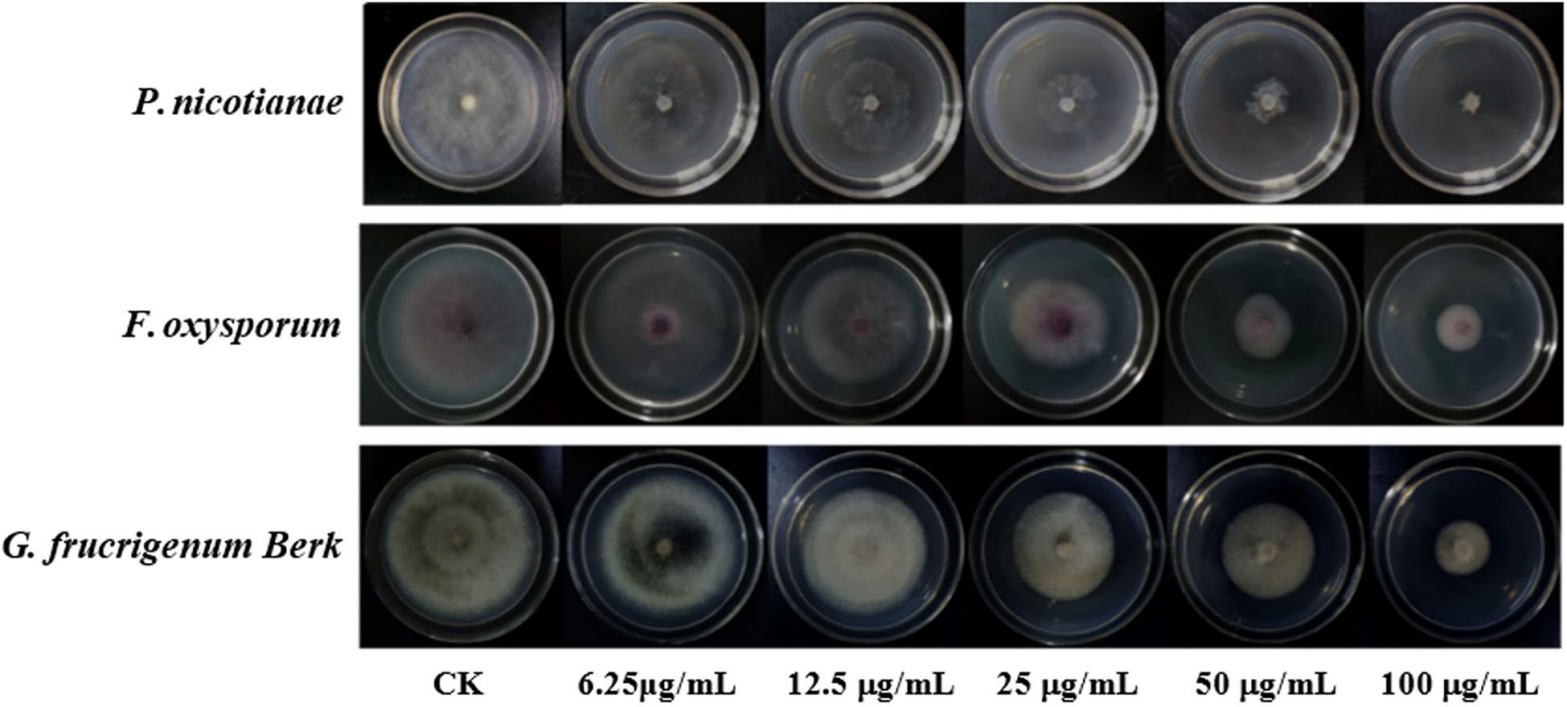
Inhibitory activity of compound 1 against mycelial growth of three plant pathogens.

Based on the results of the preliminary screening of the antimicrobial activity of the eucalyptane-type sesquiterpenoid 1, the pathogenic fungi that achieved more than 70% inhibition were selected for the determination of virulence equations (**Table 2**; **Figure 2**). As shown in **Table 2**, the eudesmane-type sesquiterpenoid 1 exhibited pronounced antifungal efficacy against the three phytopathogenic fungi, *P. nicotianae*, *F. oxysporum*, and *G. fujikuroi* Berk, with EC_50_ values of 12.56, 51.29, and 47.86 μg/mL, respectively.

**Table 2:** Inhibitory activity of compound 1 against the mycelial growth of three plant pathogens.

Plant pathogenic fungus	Toxicity regression equation	EC_50_ (μg/mL)	Correlation coefficient (R^2^)
*Phytophthora nicotianae*	y = 1.713x + 3.1137	12.56	0.979
*Fusarium oxysporum*	y = 1.3822x + 2.6427	51.29	0.9277
*Gloeosporium fructigenum* Berk	y = 1.3297x + 2.7718	47.86	0.9581

The effects of the tested compounds on three plant pathogens were repeated in triplicate.

**Figure 3:**
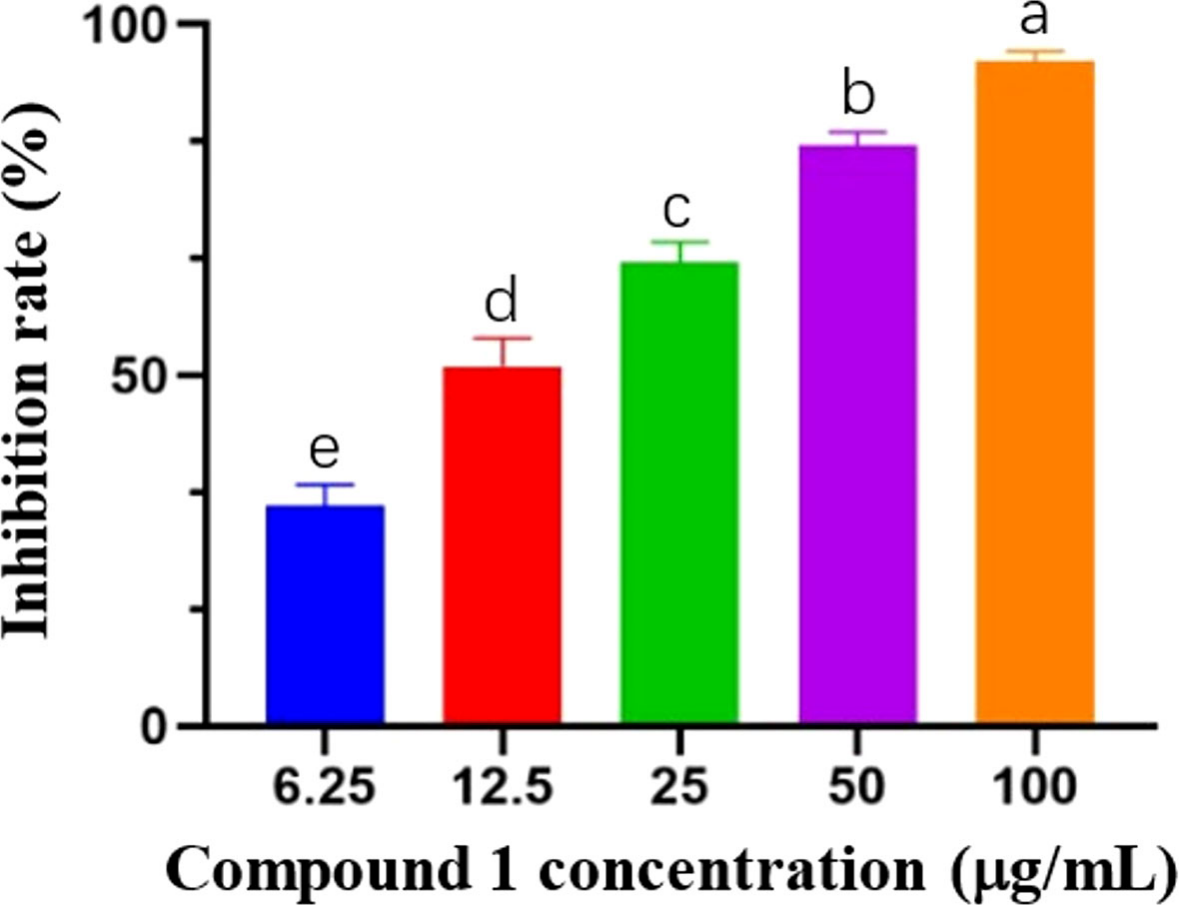
Inhibition of *Phytophthora nicotianae* by compound 1 isolated from *Laggera pterodonta*.

The results of the activity test showed that the eucalyptane-type sesquiterpenoid 1 had a good inhibitory effect against *P. nicotianae* (**Figure 3**), which shows its potential to be further developed into a novel plant-derived fungicide. Meanwhile, in order to explore the principle of the inhibition of phytopathogenic fungi by compound 1, we selected *P. nicotianae* and *F. oxysporum* as the target strains and further investigated the mechanism of action of eucalyptolide-type sesquiterpenoids in plant pathogenic fungi.

### 3.4 Determination of the MIC of compound 1 against *P. nicotianae* and *F. oxysporum*

In order to further understand the inhibitory effect of compound 1 on plant pathogenic fungi, the MIC of compound 1 against *P. nicotianae* and *F. oxysporum* was determined using the broth dilution method in a 96-well plate; the results are presented in **Table 3**. The mycelial growth of *F. oxysporum* persisted at concentrations of 0–100 μg/mL, whereas the complete inhibition of *P. nicotianae* and *F. oxysporum* was observed at 200 and 400 μg/mL, respectively. Consequently, the MICs of compound 1 against *P. nicotianae* and *F. oxysporum* were determined as 200 and 400 μg/mL, respectively.

**Table 3:** The minimum inhibitory concentration of compound **1** against *Phytophthora nicotianae* and *Fusarium oxysporum*.

Concentration (μg/mL)	0	12.5	25	50	100	200	400	800
*P. nicotianae*	+++	+++	++	++	+	−	−	−
*F. oxysporum*	+++	+++	++	++	++	+	−	−

+++, a large amount of mycelium grows; +++, a medium amount of mycelium grows; +, a small amount of mycelium grows; −, no mycelium grows.

### 3.5 Effect of compound 1 on the mycelial morphology of *P. nicotianae* and *F. oxysporum*

**Figure 4:**
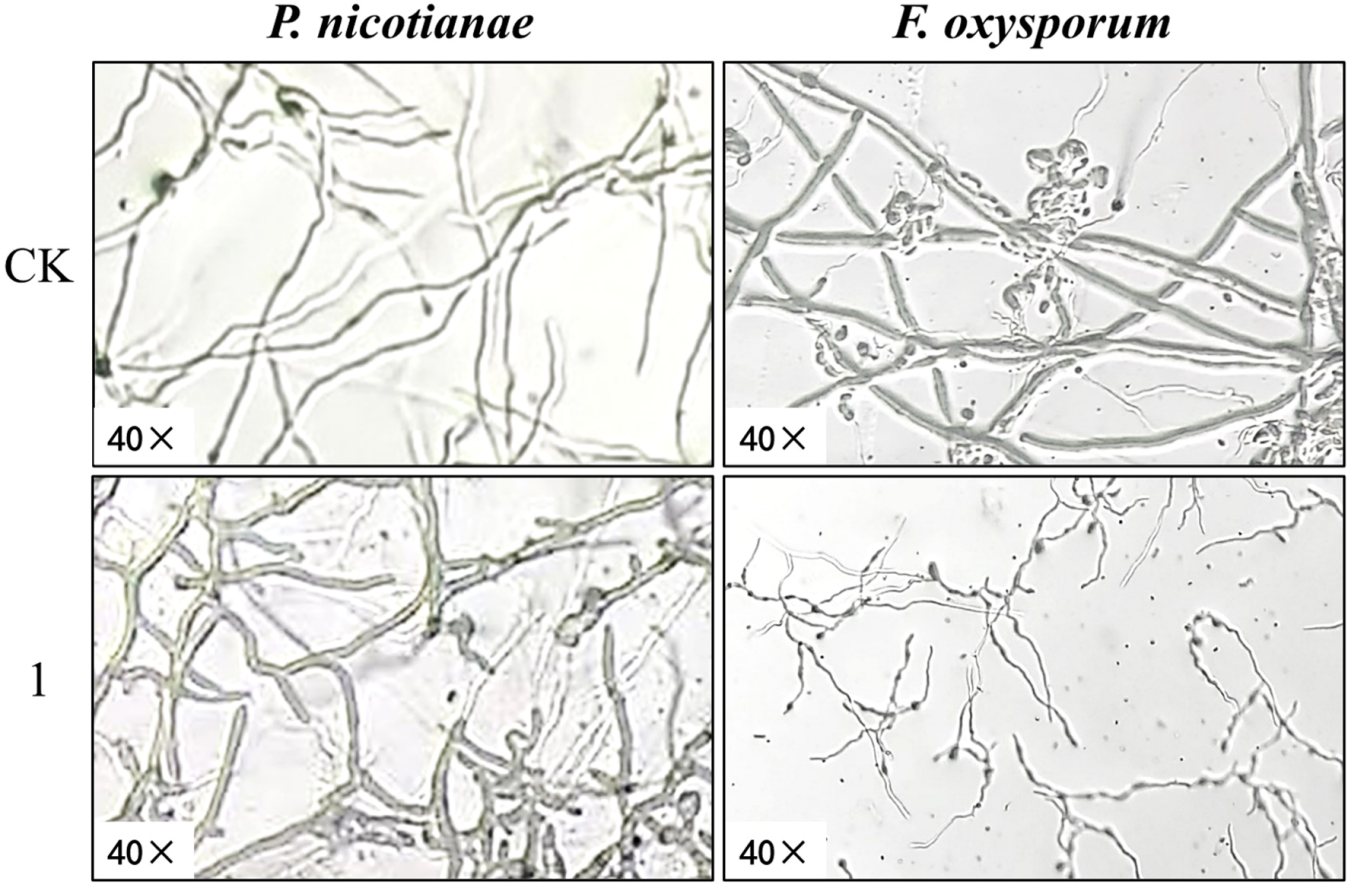
Effect of compound **1** on mycelium morphology of *Phytophthora nicotianae* and *Fusarium oxysporum* observed by light microscopy.

The EC_50_ of compound 1 for mycelial growth inhibition was applied to treat *P. nicotianae* and *F. oxysporum*, with morphological alterations assessed via light microscopy (LM) and SEM. As depicted in **Figure 4**, pronounced morphological aberrations were evident in both fungi following 24-hour exposure to compound 1 under identical LM magnification. In the control group of *P. nicotianae*, the mycelium was observed to be uniform in thickness, the surface was smooth and full, and it had formed an obvious mycelial structure. The mycelium in the treatment group was noticeably twisted and non-uniform in thickness; the mycelium was shorter and more forked, the surface was rough, and the mycelium as a whole was distributed in a deformed manner. In the *F. oxysporum* control group, the hyphae exhibited uniform morphology with robust mycelial networks, whereas treated samples displayed attenuated and fragmented hyphae, failing to establish defined mycelial structures after 24 hours. Radial expansion in the treatment group was markedly reduced compared to the controls.

**Figure 5:**
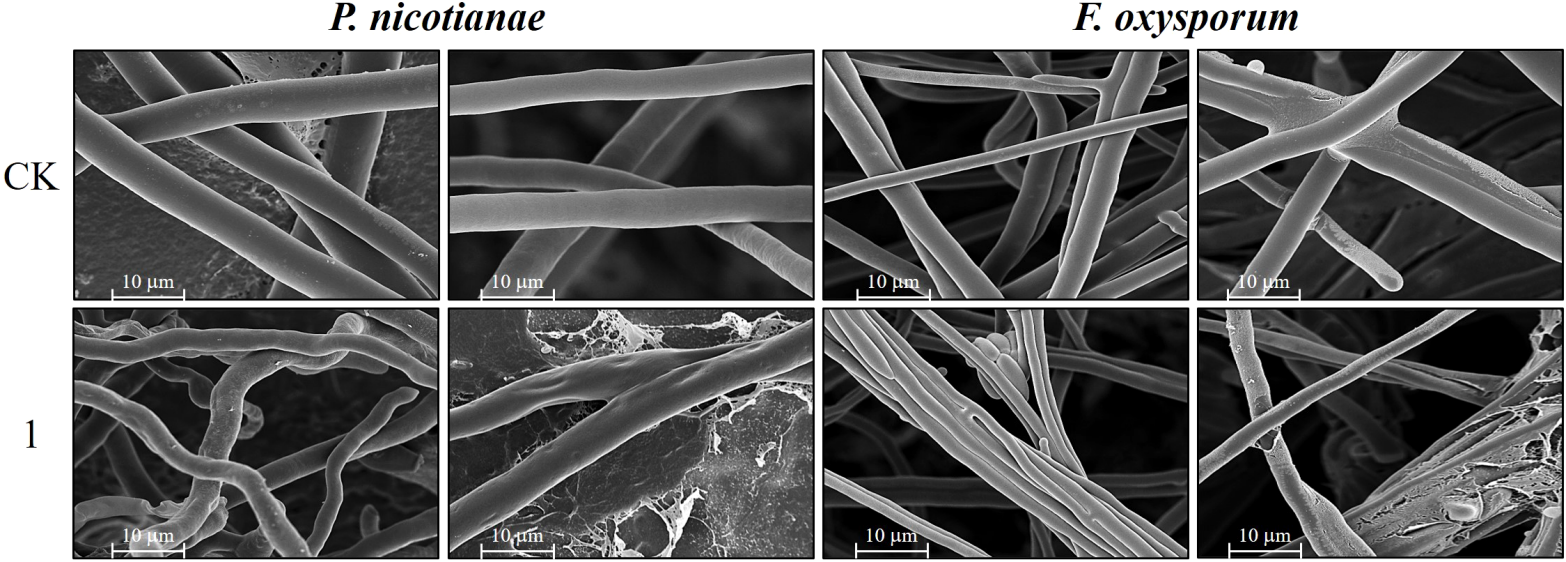
Scanning electron micrographs representing the mycelium of *Phytophthora nicotianae* and *Fusarium oxysporum* treated with compound 1.

**Figure 5** presents the SEM images of *P. nicotianae* and *F. oxysporum* mycelia treated with compound 1 at the EC_50_ concentration for mycelial growth inhibition. *P. nicotianae* control mycelium was found to be tubular, smooth, and homogeneous in morphology, and fuller, while after the action of compound 1, the surface of the fungus became rough, with white material attached, and there were deformities, such as collapse, obvious loss of water, crumpling, and bending and folding, which was in line with the phenomenon observed under the light microscope. The *F. oxysporum* control mycelium surface was also relatively smooth, and the mycelium and spores were very full, while in the compound **1** treatment, the most obvious changes were that the diameter of the mycelium became thinner and shorter, the spores became smaller, and the growth rate lagged. This phenomenon is also consistent with the phenomenon observed during light microscopy imaging.

### 3.6 Effect of compound 1 on the membrane integrity of fungal cells

**Figure 6:**
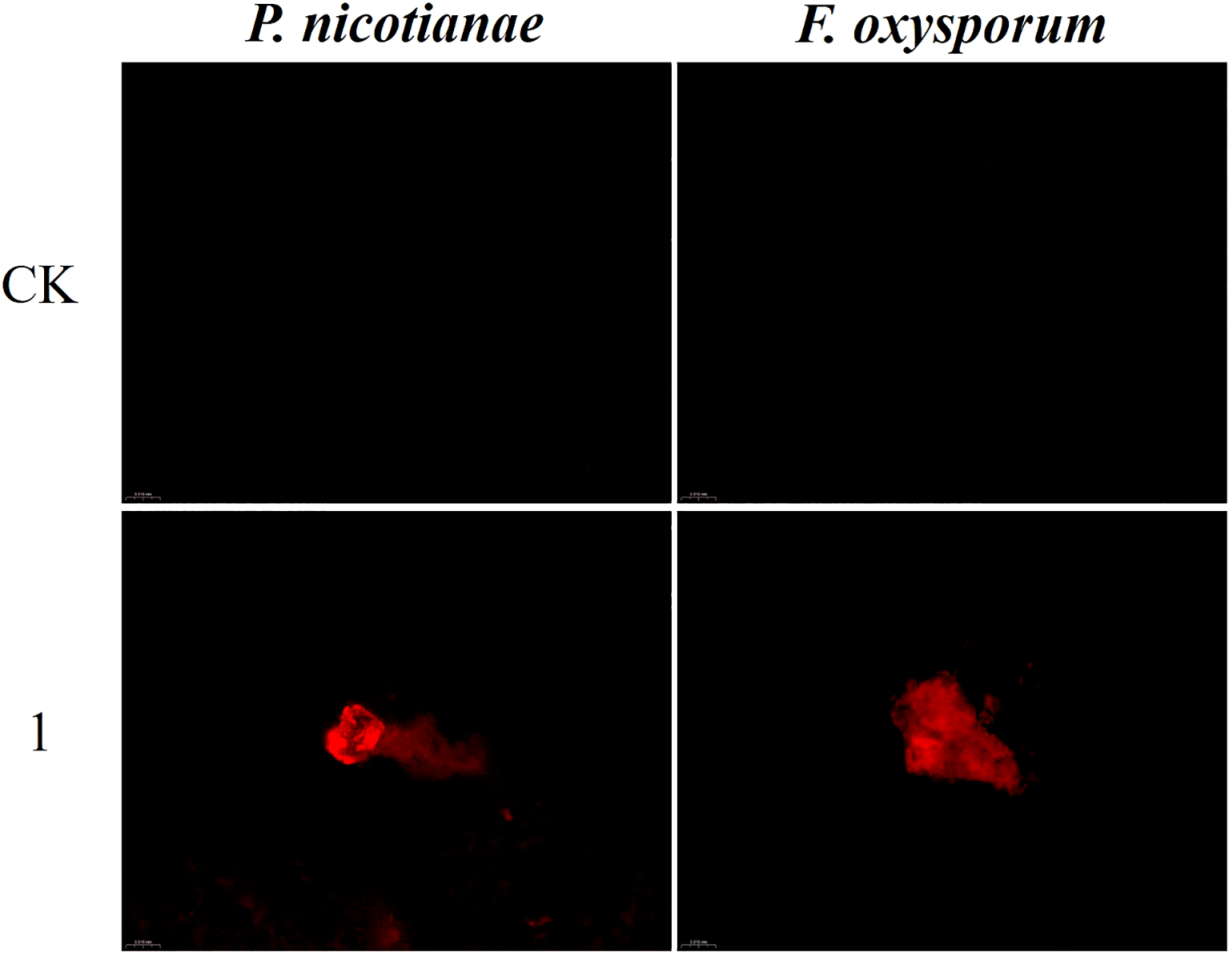
Effects of compound 1 on the cell membrane integrity of *Phytophthora nicotianae* and *Fusarium oxysporum*. CK represents the control.

PI, a membrane-impermeant nucleic acid stain, fluoresces upon binding to intracellular DNA/RNA, serving as a marker for compromised membrane integrity. Late-stage apoptotic cells exhibit PI uptake, reflecting increased membrane permeability. As shown in **Figure 6**, no discernible PI fluorescence was observed in untreated controls under dark-field excitation. In contrast, compound 1-treated *P. nicotianae* and *F. oxysporum* exhibited pronounced red fluorescence, indicating membrane disruption and elevated permeability. These findings suggest that compound 1 induces structural damage to fungal cell membranes, likely mediating its antifungal activity. Collectively, the data support the hypothesis that the eudesmane-type sesquiterpenoid 1 exerts antifungal effects by compromising membrane integrity.

The current understanding of the antifungal mechanisms of plant-derived agents remains limited. Extant studies have suggested that these compounds primarily disrupt fungal physiology through three pathways: 1) compromising cell ultrastructure, 2) perturbing metabolic homeostasis, and 3) impairing bioenergetic processes (Zhao et al., 2022). For example, Li et al. (2017) found that tea tree oil could damage the cell wall and cell membrane of *B. cinerea* through increased membrane permeability, which ultimately affects the growth of *B. cinerea*. Furthermore, numerous studies have demonstrated that antifungal agents disrupt fungal cell walls and membrane integrity, inducing membrane hyperpermeability and thereby suppressing hyphal proliferation (Ou et al., 2019; He et al., 2018). Regarding the effects of fungal inhibitors on the cellular metabolism of fungal cells, Chen et al. (2018) found that curcumin could inhibit the activity of *Fusarium graminearum* Schw-related enzymes and affect tRNA synthesis and glucose metabolism using proteomics. Additionally, several reports have provided comprehensive information about the effect of bacteriostatic active substances on the cellular energy of fungi. For instance, emerging evidence demonstrates that curcumin perturbs both material metabolism and energy homeostasis in the phytopathogen *F. graminearum* Schw (Chen et al., 2018).

Based on the observed morphological aberrations and compromised membrane integrity in *P. nicotianae* and *F. oxysporum* mycelia treated with compound 1, we hypothesized that its antifungal activity involves dual mechanisms: 1) direct disruption of membrane architecture, increasing permeability and triggering leakage of essential metabolites, thereby altering hyphal morphology; and 2) interference with metabolic pathways critical for fungal proliferation, leading to growth arrest. While these findings provide preliminary insights, the inhibition mechanism of compound 1 remains incompletely resolved. Antifungal agents often exhibit multifactorial and polypharmacological modes of action, necessitating further investigation to delineate specific molecular targets and pathways. Nevertheless, the data collectively substantiate compound 1 as a promising candidate for the development of novel plant-derived fungicides, offering both a theoretical framework and methodological precedents for future studies.

### 3.7 ADMET prediction of active compounds

The ADMET property of a compound denotes the five key notes as absorption, distribution, metabolism, excretion, and toxicity of a compound in an organism (Deng et al., 2012). The main indicators are the blood–brain barrier (BBB), human intestinal absorption (HIA), Caco-2 permeability (CCP), Ames mutagenicity (ATT), carcinogenicity, and cytochrome (CYP2D6). ADMET prediction of compounds helps to evaluate the drug capability and safety of compounds in the early stages of drug development, which not only helps to increase the success rate of drug development but also reduces the cost of drug development.

Based on antifungal activity screening results, compounds 1, 3, and 6—exhibiting selective antifungal efficacy against key phytopathogens—were prioritized for ADMET profiling (**Table 4**). Computational predictions indicate favorable intestinal absorption and bioavailability for all three compounds, with demonstrable permeability across intestinal epithelial barriers. Notably, none exhibited mutagenic or carcinogenic liabilities, suggesting low toxicological risks. In addition, as compared to compounds 1 and 3 (which were able to cross the blood–brain barrier), compound 6 did not easily cross the blood–brain barrier. However, the difference in their structures lies in the presence or absence of hydroxyl groups on the backbone, so we speculate that the presence or absence of backbone hydroxyl groups had a significant effect on the blood–brain barrier passage ability of eucalyptus-type sesquiterpenoids. These findings indicate that all three compounds have good ADMET properties and have characteristics that can be developed into novel drugs.

**Table 4:** ADMET prediction results for compounds.

Compound	BBB	HIA	CCP	ATT	Carcinogenicity	CYP2D6 inhibitor
1	0.7250+	0.9950+	0.7249+	None	None	Non-inhibitor
3	0.7000+	0.9946+	0.8665+	None	None	Non-inhibitor
6	0.5000−	0.9956+	0.7609+	None	None	Non-inhibitor

BBB “+” in the table indicates that the drug molecules can easily pass through the blood–brain barrier. BBB “−” indicates that the drug molecules cannot easily pass through the blood–brain barrier; the closer the value is to 1, the better the permeability of the drug to the blood–brain barrier. HIA “+” indicates that the drug molecules can be absorbed or assimilated through human intestines; the closer the value is to 1, the better the absorption through the intestines. CCP “+” indicates that the drug molecules can easily penetrate the human intestinal cell line; the closer the value is to 1, the better the permeability of CCP. “None” indicates that the compound is not mutagenic or carcinogenic. ADMET, absorption, distribution, metabolism, excretion, and toxicity; BBB, blood–brain barrier; HIA, human intestinal absorption; CCP, Caco-2 permeability; ATT, Ames mutagenicity.

## 4 Conclusions

In this study, six eudesmane-type sesquiterpenoids isolated from *L. pterodonta* were evaluated for antifungal activity. The compounds exhibited varying inhibitory effects on six phytopathogens (*P. nicotianae*, *F. oxysporum*, *G. fujikuroi* Berk., *A. alternata*, *C. fructicola*, and *B. cinerea*). Compound 1 demonstrated potent inhibition against *P. nicotianae*, *F. oxysporum*, and *G. fujikuroi* (94.64%, 72.55%, and 70.46% inhibition, respectively), with EC_50_ values of 12.56, 51.29, and 47.86 μg/mL, respectively, and MICs of 200 and 400 μg/mL for *P. nicotianae* and *F. oxysporum*, respectively. Compound 3 inhibited *F. oxysporum* (58.82%), while compound 6 suppressed *P. nicotianae* (73.92%). Compound 1 induced severe morphological aberrations and compromised membrane integrity in *P. nicotianae* and *F. oxysporum*, elevating permeability and disrupting cellular homeostasis. All three bioactive compounds exhibited favorable ADMET profiles, underscoring their potential as eco-friendly, plant-derived fungicides, which may also serve as the basis for the development of new fungicides.

## Data Availability

The original contributions presented in the study are included in the article/**Supplementary Material**. Further inquiries can be directed to the corresponding authors.

## References

[B1] Bhandari S. Yadav P. K. Sarhan A. T. (2021). Botanical fungicides; current status, fungicidal properties and challenges for wide scale adoption: a review. J. Rev. In Food And Agriculture. 2, 63–68. doi: 10.26480/rfna.02.2021.63.68

[B2] Bo T. Liu M. Zhong C. Zhang Q. Su Q. Z. Tan Z. L.. (2014). Metabolomic analysis of antimicrobial mechanisms of ϵ-poly-L-lysine on Saccharomyces cerevisiae. J. J. Agric. Food Chem. 62, 4454–4465. doi: 10.1021/jf500505n, PMID: 24735012

[B3] Cao C. M. Shen W. Z. Li Y. L. Wang H. Gao M. Y. (2011). Effects and mechanisms of 3,5-dihydroxy-6,7,3′,4′-tetramethoxyflavone from the traditional Chinese medicine Laggera pterodonta on apoptosis of human nasopharyngeal carcinoma CNE cells. J. Adv. Biochem. Biophysics. 38, 254–261. doi: 10.3724/SP.J.1206.2010.00539

[B4] Chen G. Y. Chen X. B. Liu G. M. (2012). Study on the chemical composition of Stinking Lingdan. J. Anhui Agric. Science. 40, 2022–2023. doi: 10.13989/j.cnki.0517-6611.2012.04.126

[B5] Chen C. Long L. Zhang F. Chen Q. Chen C. Yu X. R.. (2018). Antifungal activity, main active components and mechanism of Curcuma longa extract against Fusarium graminearum. J. PloS One 13, 194284. doi: 10.1371/journal.pone.0194284, PMID: 29543859 PMC5854386

[B6] Cruz R. Martinez R. M. (1982). Stereoselective total synthesis of (±) – isocostic and (±)-3-Oxoisocostic acids. Aust. J. Chem. 35, 451–456. doi: 10.1071/CH9820451

[B7] Deng Z. T. Qi X. Li J. (2012). Research progress on high-throughput screening technology for *in vitro* ADMET of innovative drugs. J. Adv. Modern Biomedicine. 12, 1176–1178. doi: 10.13241/j.cnki.pmb.2012.06.051

[B8] Ding L. Li H. C. Wang B. Q. Liu G. A. (2017). Bacteriostatic activities of the major secondary metabolites of four species of Isodon amethystoides from Gansu. J. J. Northwest Normal University (Natural Sci. Edition). 53, 82–87. doi: 10.16783/j.cnki.nwnuz.2017.02.015

[B9] Gao T. T. Yang Y. H. Yang N. Li B. Jin H. Tao K.. (2019). Extraction and isolation of locust-killing active components of Laggera pterodonta. J. J. Sichuan University. 56, 142–148. doi: 10.3969/j.issn.0490-6756.2019.01.025

[B10] He J. Wu D. Zhang Q. Chen H. Li H. Y. Han Q. H.. (2018). Efficacy and mechanism of cinnamon essential oil on inhibition of Colletotrichum acutatum isolated from ‘Hongyang’ Kiwifruit. J. Front. Microbiol. 9, 1288. doi: 10.3389/fmicb.2018.01288, PMID: 29967599 PMC6015887

[B11] Isela S. F. Everardo L. R. Georgina R. L. Alejandrina M. G. M. Arturo V. G. (2015). Influence of culture media on biofilm formation by Candida species and response of sessile cells to antifungals and oxidative stress. J. BioMed. Res. Int. 2015, 783639. doi: 10.1155/2015/783639, PMID: 25705688 PMC4331161

[B12] Kalliopi S. Demosthenis I. Apostolos S. Anita B. Athanassios G. Haralambos E. (2017). Use of costic acid, a natural extract from Dittrichia viscosa, for the control of Varroa destructor, a parasite of the European honey bee. J. Beilstein J. Organic Chem. 13, 952–959. doi: 10.3762/bjoc.13.96, PMID: 28684976 PMC5480341

[B13] Liu Q. J. Fu L. X. Zhang Y. Li F. C. Yu H. Li J. L.. (2022). Fumigant activity and chemical composition analysis of essential oil of Dodonaea viscosa seeds against five plant pathogenic fungi. J. South. J. Agriculture. 53, 1935–1943. doi: 10.3969/j.issn.2095-1191.2022.07.016

[B14] Li J. L. Li F. C. Wu G. X. Gui F. R. Li H. M. Xu L. L.. (2023). Acetylcholinesterase inhibitory activity of sesquiterpenoids isolated from Laggera pterodonta. J. Front. Plant Science. 14, 1074184–1074184. doi: 10.3389/fpls.2023.1074184, PMID: 36844064 PMC9950556

[B15] Li Y. Shao X. Xu J. Wei Y. Y. Xu F. (2017). Tea tree oil exhibits antifungal activity against Botrytis cinerea by affecting mitochondria. J. Food Chem. 234, 62–67. doi: 10.1016/j.foodchem.2017.04.172, PMID: 28551268

[B16] Li S. H. Zhao Q. Liu F. Lei K. J. (2013). Identification and antioxidant activity of flavonoids in Laggera pterodonta. J. Modern Food Sci. Technology. 29, 1213–1216. doi: 10.13982/j.mfst.1673-9078.2013.06.013

[B17] Lu P. W. Chen L. J. Li W. (2014). Chemical constituents from Laggera pterodonta. J. J. Chin. medicinal materials. 37, 816–819. doi: 10.13863/j.issn1001-4454.2014.05.022, PMID: 25335290

[B18] Luo J. M. Zhan X. Y. Wu J. R. Liu L. Hong Y. T. Zhang D. H.. (2022). Screening of plant extracts for fungus inhibitory activity against Colletotrichum gloeosporioides. J. Chin. J. Biol. Control. 38, 852–859. doi: 10.16409/j.cnki.2095-039x.2022.04.009

[B19] Ou Y. Q. L. Duan X. F. Li L. Tao N. G. (2019). Cinnamaldehyde exerts its antifungal activity by disrupting the cell wall integrity of Geotrichumcitri-aurantii. J. Front. Microbiol. 10, 55. doi: 10.3389/fmicb.2019.00055, PMID: 30761105 PMC6364577

[B20] Prastiwi S. Wagiyana W. Y. Alfarisy F. K. (2023). Compatibility studies of entomopathogenic fungi and botanical pesticide for controlling *Spodoptera exigua.* J. E3S Web Conferences. 373, e100345. doi: 10.1051/e3sconf/202337307006

[B21] San J. F. Castellano G. Marco J. (1990). Sesquiterpene lactones from Artemisia herba-alba. J. Phytochemistry. 29, 541–545. doi: 10.1016/0031-9422(90)85114-U

[B22] Saravana G. (2022). Plants and phytochemical activity as botanical pesticides for sustainable agricultural crop production in India-MiniReview. J. J. Agric. Food Res. 9, e100345. doi: 10.1016/J.JAFR.2022.100345

[B23] Wang Y. X. (2019). Overview of the research on Laggera pterodonta, a commonly used folk medicine in Yunnan. J. Yunnan J. Traditional Chin. Med. 40, 74–75. doi: 10.16254/j.cnki.53-1120/r.2019.07.031

[B24] Wang H. Li P. (2022). Determination of virulence of different fungicides against Venturia inaequalis. J. Modern Pesticides. 21, 55–57. doi: 10.3969/j.issn.1671-5284.2022.06.010

[B25] Wang C. Y. Lu C. S. Chang F. H. (2020). Research on the fungus inhibition of Alternaria panax Whetz by effective parts of Sophorae Flavescentis Radix. J. South. Agriculture. 14, 159–183. doi: 10.19415/j.cnki.1673-890x.2020.29.076

[B26] Wei J. C. Chen Y. Wei Z. X. Ye X. Que Z. L. Yu Y. H.. (2017). Progress of research on the pharmacology and quality analysis of hexandra chinensis. J. Guangzhou Chem. Industry. 45, 1–2. doi: 10.3969/j.issn.1001-9677.2017.02.001

[B27] Xie Y. Q. Fan W. T. Chen X. Q. Li R. T. Zhang Z. J. (2021). Chemical constituents from Laggera pterodonta. J. Biochem. Systematics Ecology. 94, 104222. doi: 10.1016/j.bse.2020.104222

[B28] Xie W. D. Weng C. W. Shen T Gao X. (2011). Sesquiterpenoids from aster himalaicus. J. Chem. Nat. Compd+. 47, 309–310. doi: 10.1007/s10600-011-9916-2

[B29] Yang X. R. Liao Y. F. Zhao P. F. Qin Y. M. Ou Y. X. H. (2022). Progress of research on plant-derived pesticides and their development and utilization. J. South. Agriculture. 16, 33–36. doi: 10.19415/j.cnki.1673-890x.2022.11.010

[B30] Ye X. Qu Z. Qi Z. Handong S. Françoise G. Yu Z.. (2003). Eudesmane derivatives from Laggera pterodonta. J. Fitoterapia. 74, 459–463. doi: 10.1016/S0367-326X(03)00106-0, PMID: 12837361

[B31] Ying Q. X. Yue D. L. Yan L. Q. (2006). Eudesma-5,12-dien-13-oic acid from Laggera pterodonta. Acta Crystallogr E. 62, o1844–o1845. doi: 10.1107/S1600536806012529

[B32] Zhang T. Z. Li J. H. Shen A. Li S. Z. (2023). Bacteriostatic and preservative effects of multimethoxy flavonoid extracts from citrus peels on red grapes. J. Food Industry Sci. Technol. 44, 143–150. doi: 10.13386/j.issn1002-0306.2022090038

[B33] Zhao Y. T. Wang X. E. Zhao Y. Pan T. Liu L. M. (2022). Current status and prospect of research on fungus inhibiting active ingredients and inhibitory mechanism of plant sources. J. Agric. Technology. 42, 26–29. doi: 10.19754/j.nyyjs.20220830007

