# Supplementary material for: Fungal inhibitory activity of sesquiterpenoids isolated from Laggera pterodonta
Source: Front Plant Sci. 2025 Jul 16;16:1586984. doi: 10.3389/fpls.2025.1586984 (PMC12307368; doi:10.3389/fpls.2025.1586984)
Supplement: Supplementary file 1 [file DataSheet1.docx]

Supplementary Material

**Figure S1** ^1^H NMR spectrum of compound **1** A in CDCl_3_

**Figure S2** ^13^C NMR spectrum of compound **1** in CDCl_3_

**Figure S3** ^1^H NMR spectrum of compound **2** in CD_3_OD

**Figure S4** ^13^C NMR spectrum of compound **2** CD_3_OD

**Figure S5** ^1^H NMR spectrum of compound **3** in CD_3_OD

**Figure S6** ^13^C NMR spectrum of compound **3** in CD_3_OD

**Figure S7** ^1^H NMR spectrum of compound **4** in CDCl_3_

**Figure S8** ^13^C NMR spectrum of compound **4** in CDCl_3_

**Figure S9** ^1^H NMR spectrum of compound **5** in CDCl_3_

**Figure S10** ^13^C NMR spectrum of compound **5** in CDCl_3_

**Figure S11** ^1^H NMR spectrum of compound **6** in CDCl_3_

**Figure S12** ^13^C NMR spectrum of compound **6** inCDCl_3_


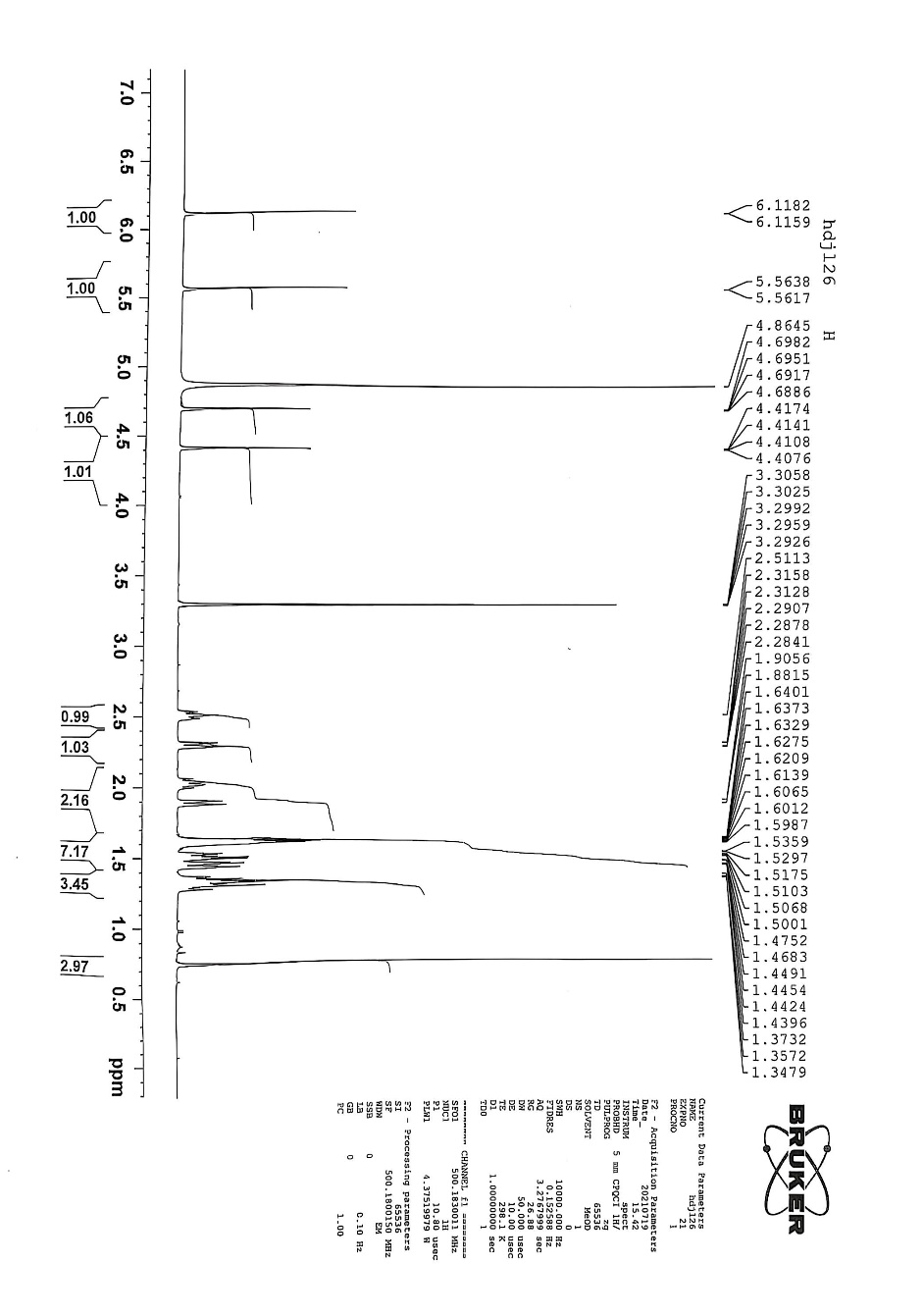


**Figure S1** ^1^H NMR spectrum of compound **1** A in CDC_l3_


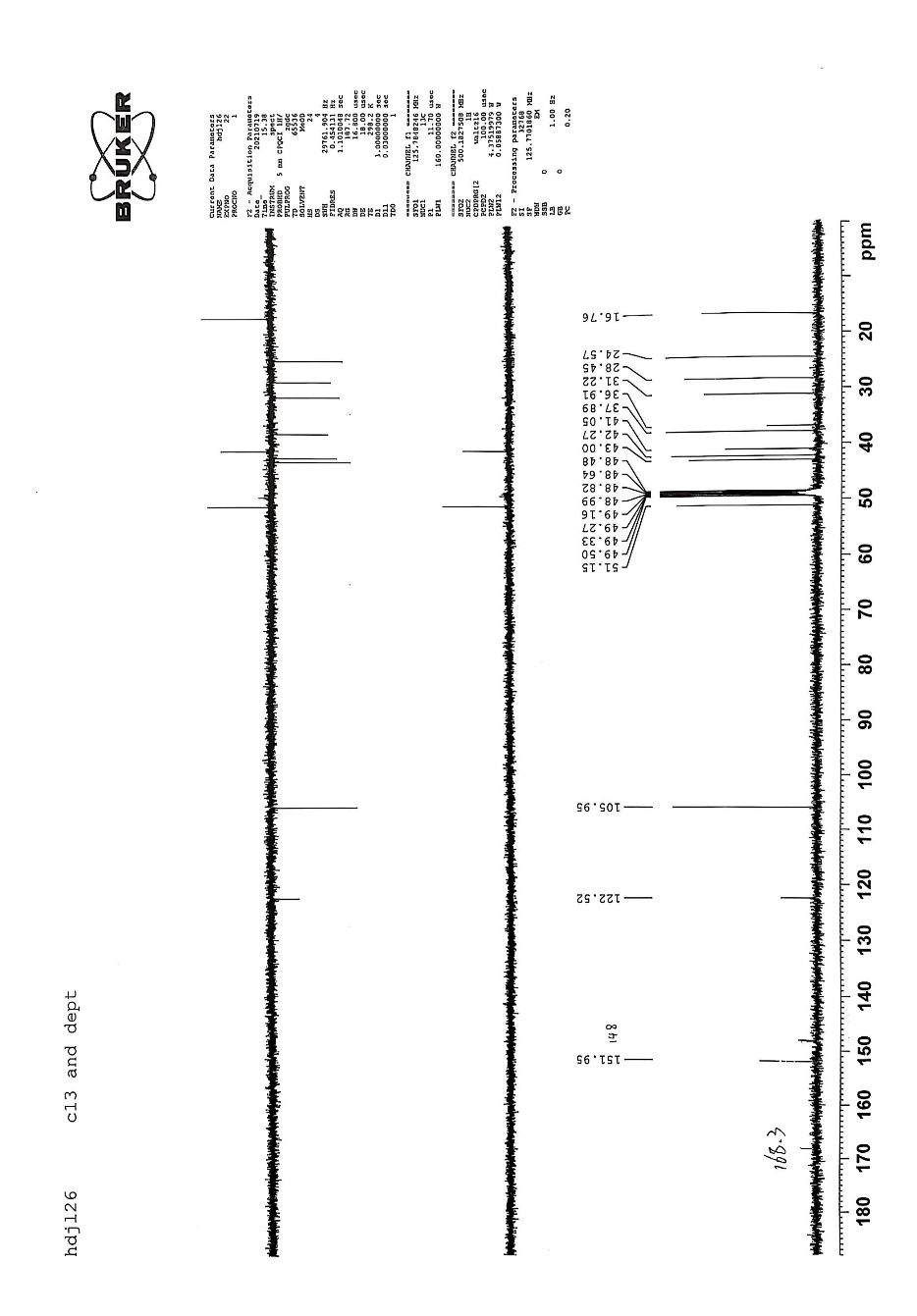


**Figure S2** ^13^C NMR spectrum of compound **1** in CDC_l3_


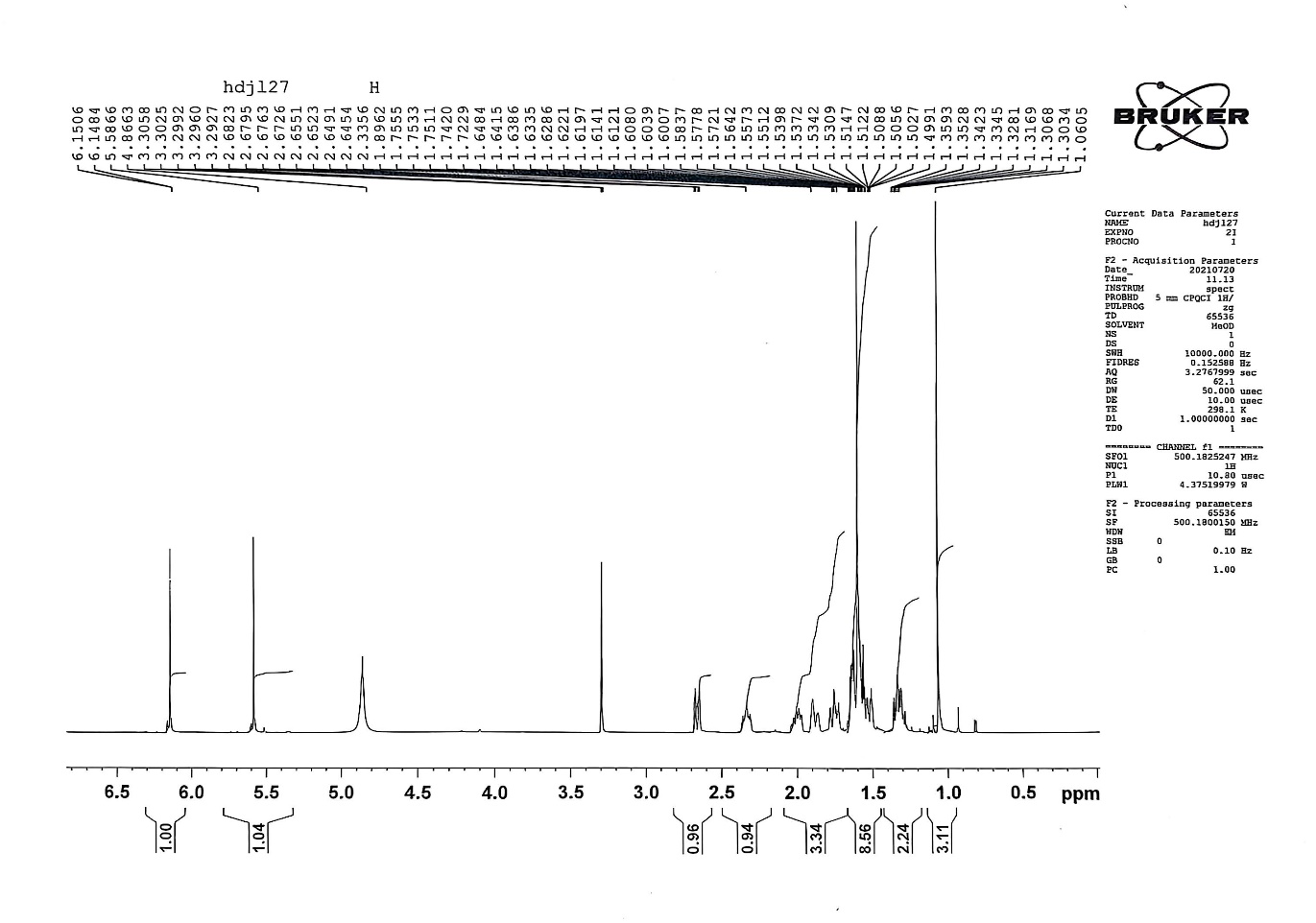


**Figure S3** ^1^H NMR spectrum of compound **2** in CD_3_OD


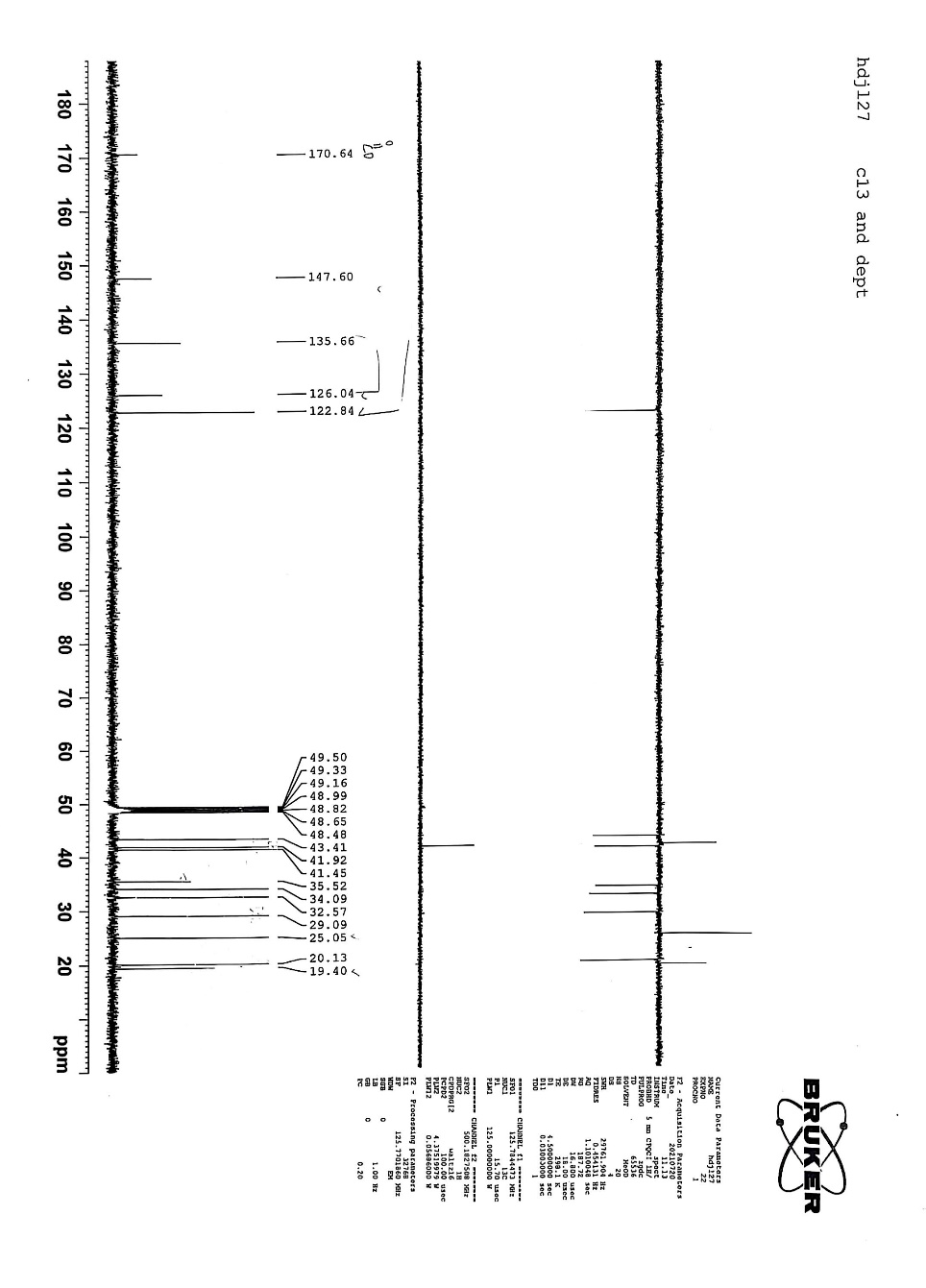


**Figure S4** ^13^C NMR spectrum of compound **2** CD_3_OD


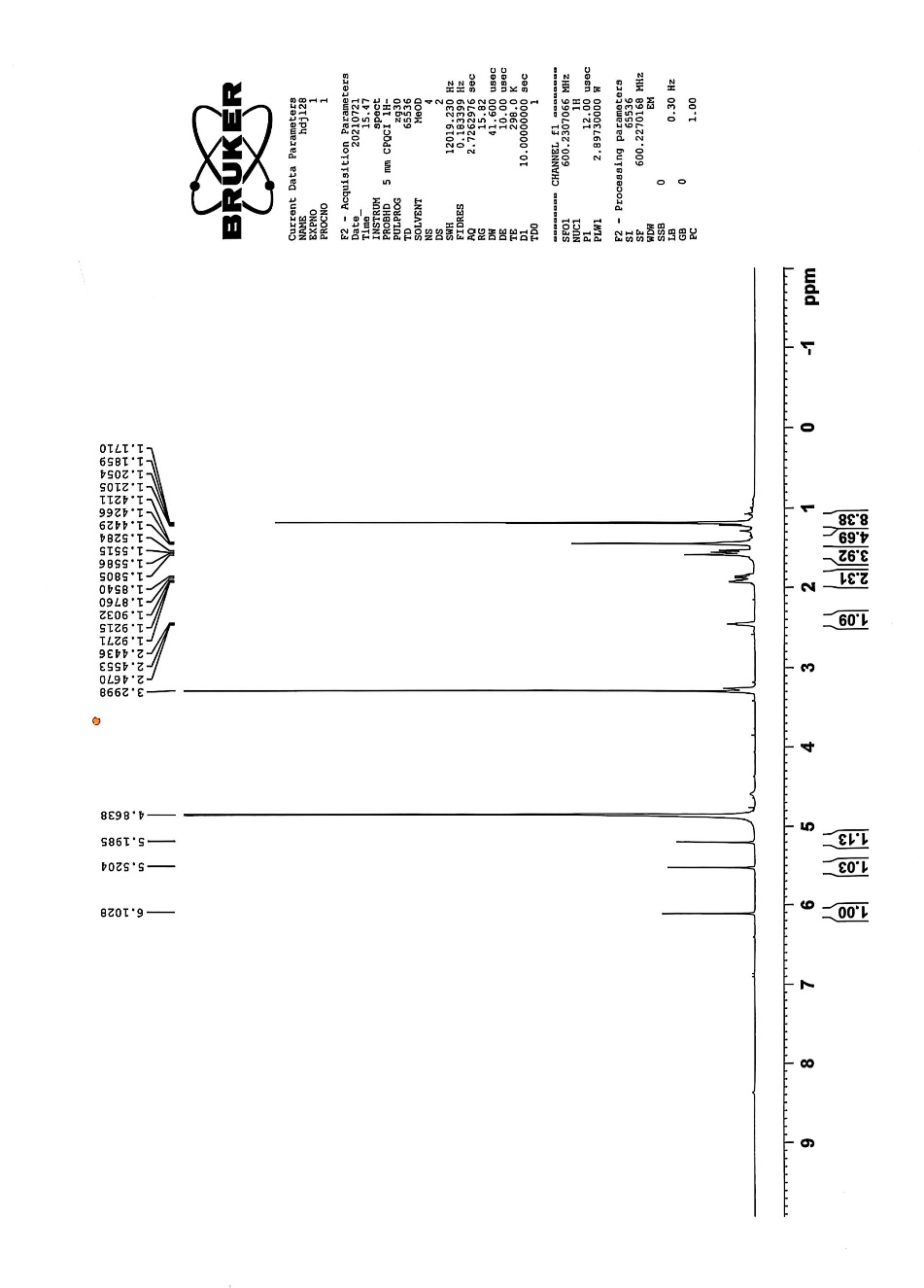


**Figure S5** ^1^H NMR spectrum of compound **3** in CD_3_OD


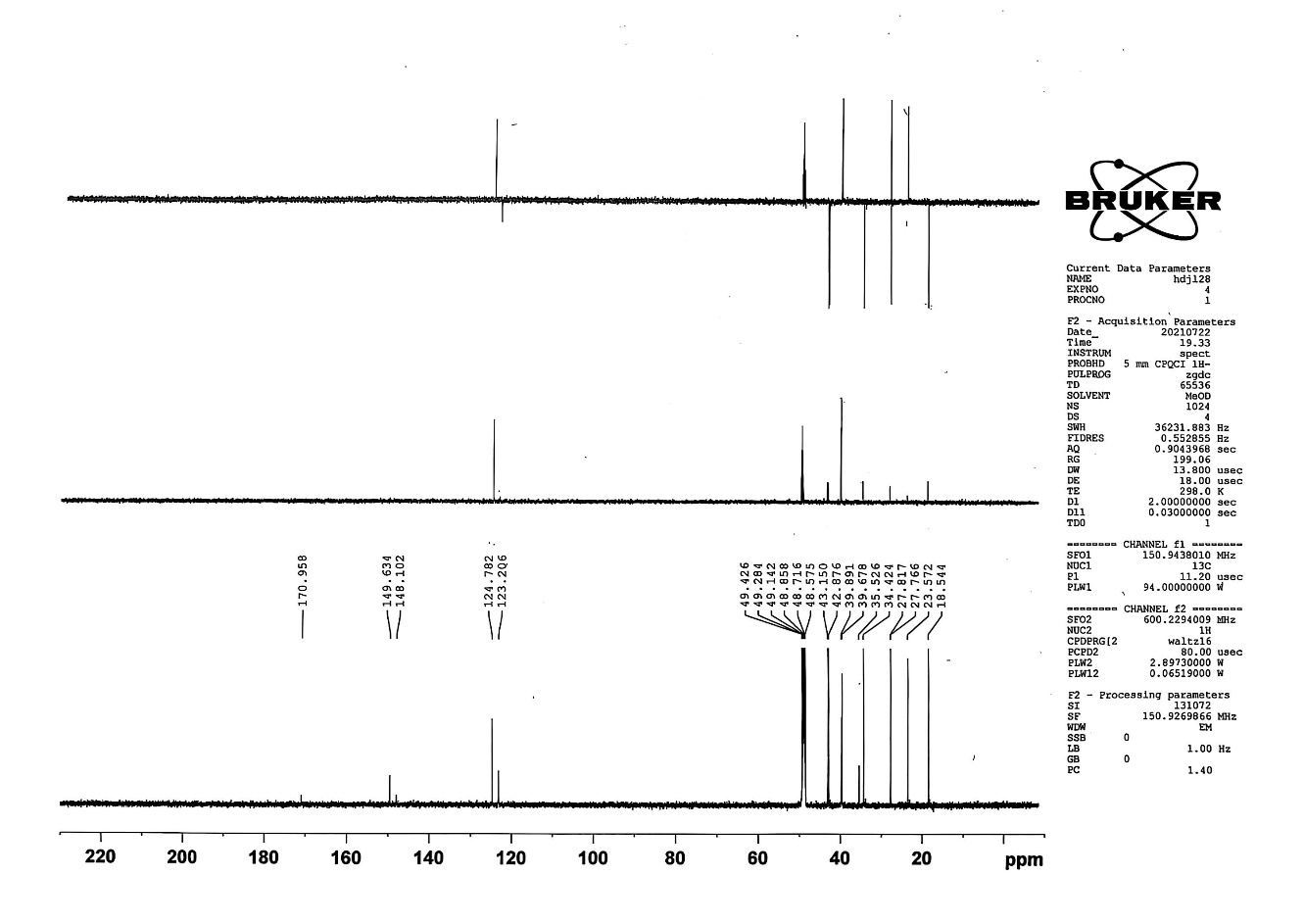


**Figure S6** ^13^C NMR spectrum of compound **3** in CD_3_OD


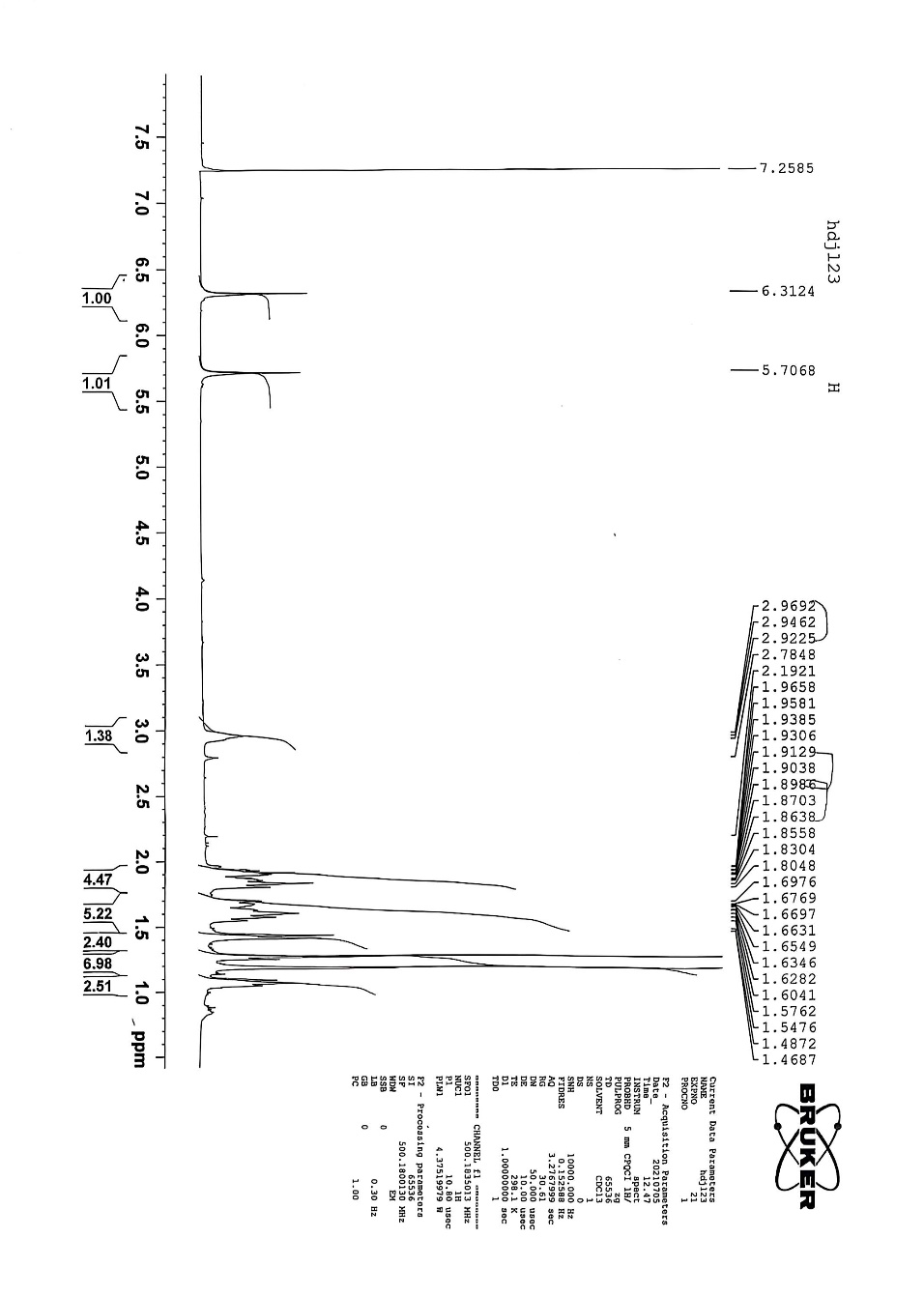


**Figure S7** ^1^H NMR spectrum of compound **4** in CDC_l3_


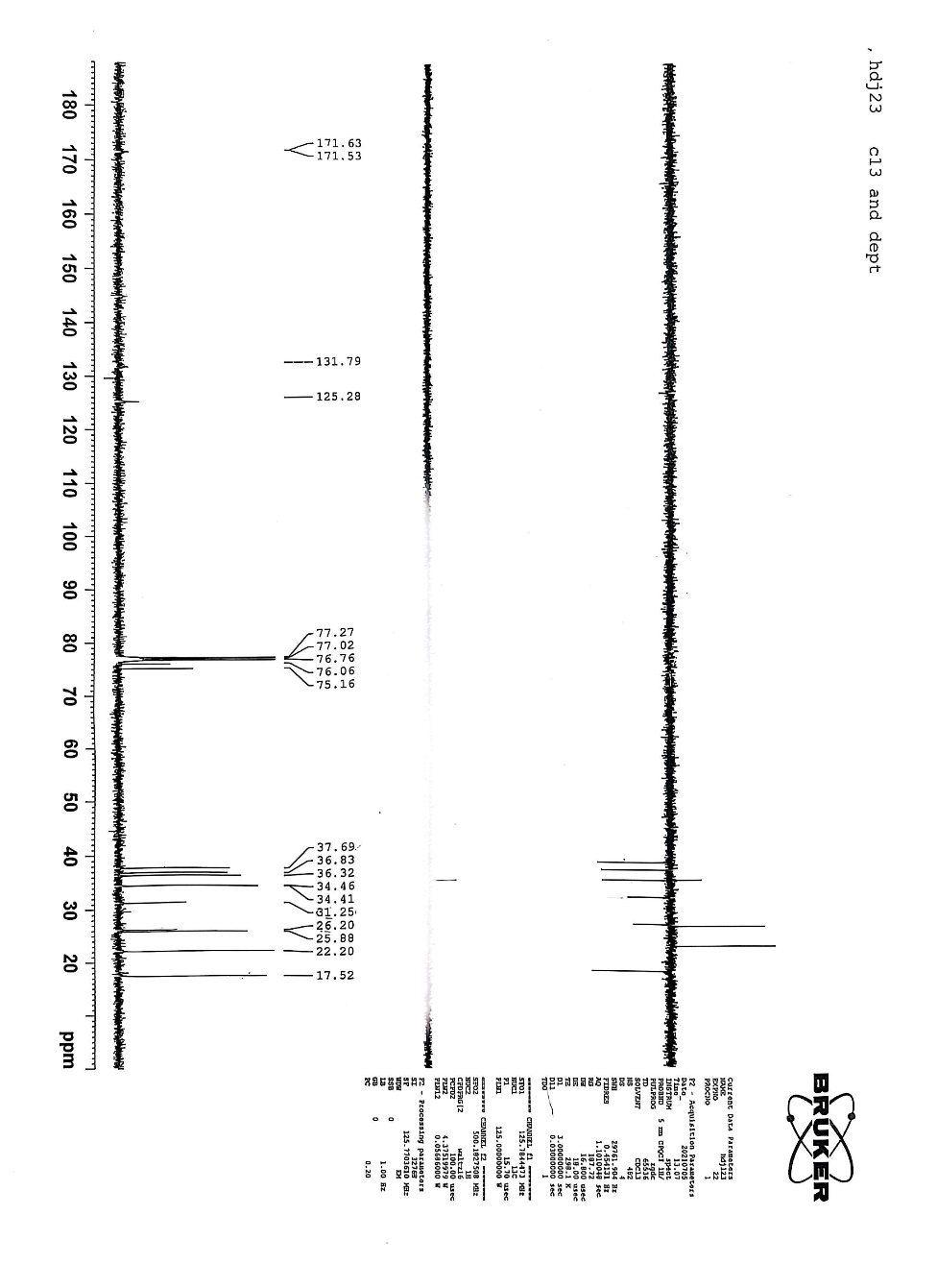


**Figure S8** ^13^C NMR spectrum of compound **4** in CDC_l3_


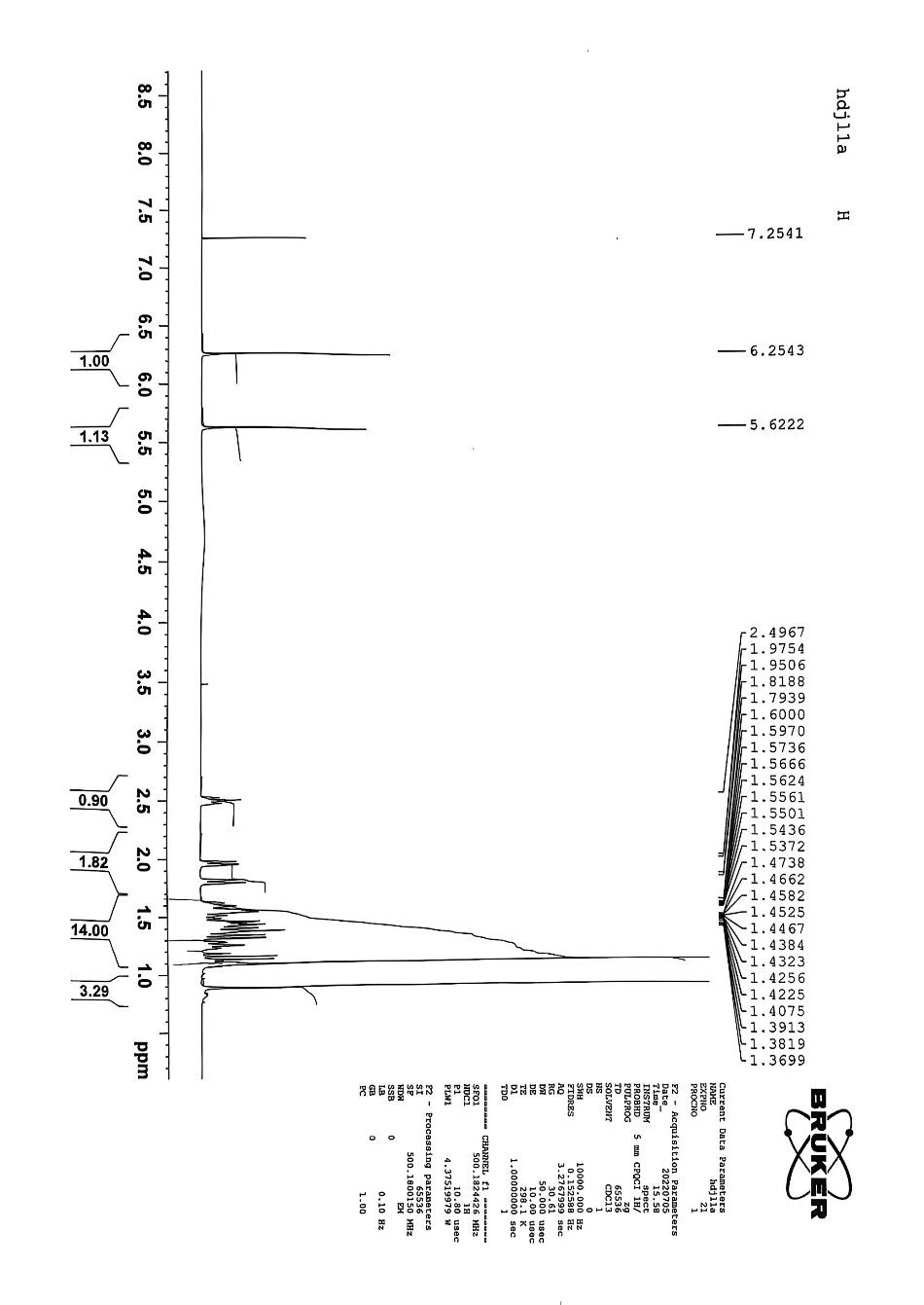


**Figure S9** ^1^H NMR spectrum of compound **5** in CDC_l3_


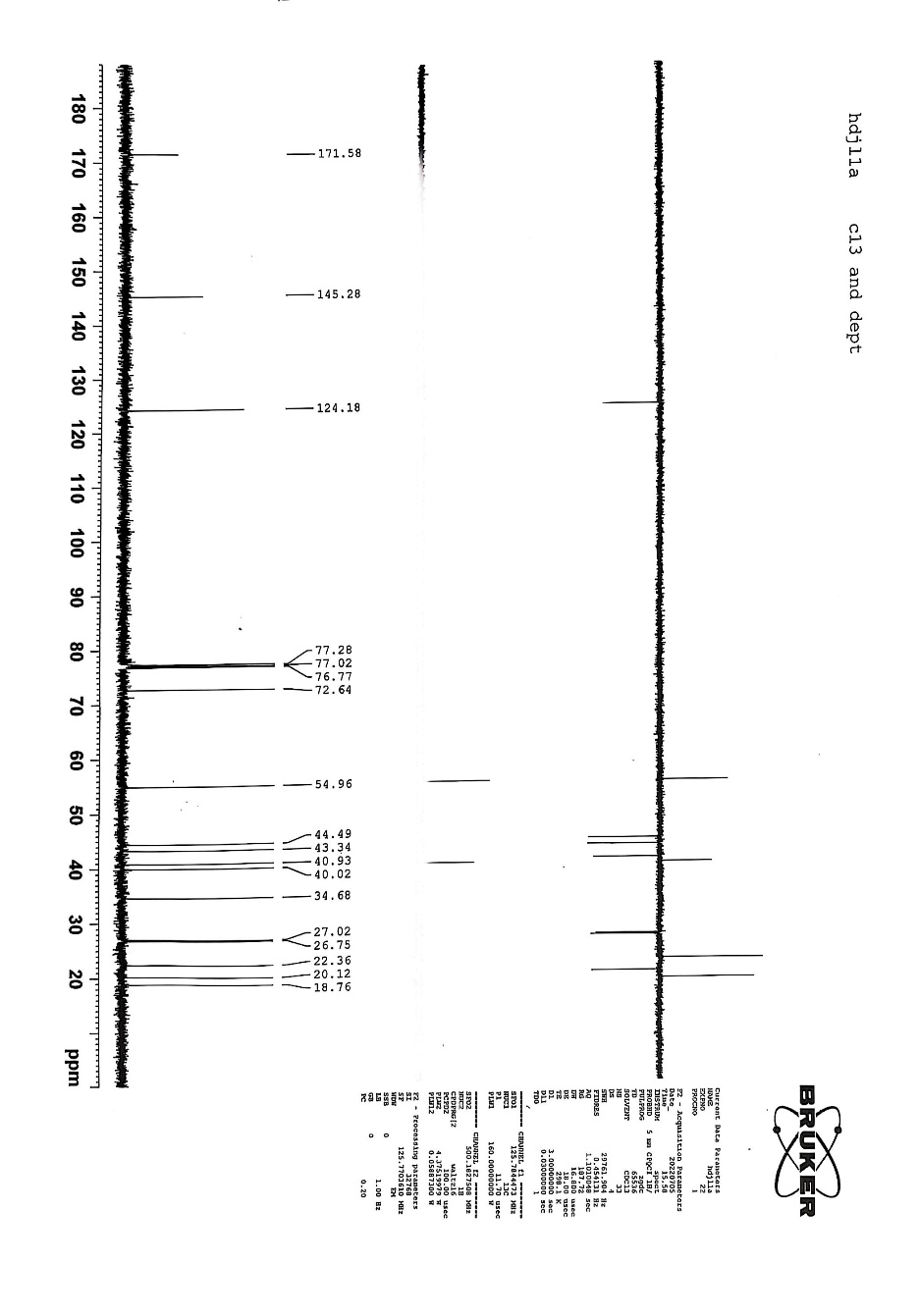


**Figure S10** ^13^C NMR spectrum of compound **5** in CDC_l3_


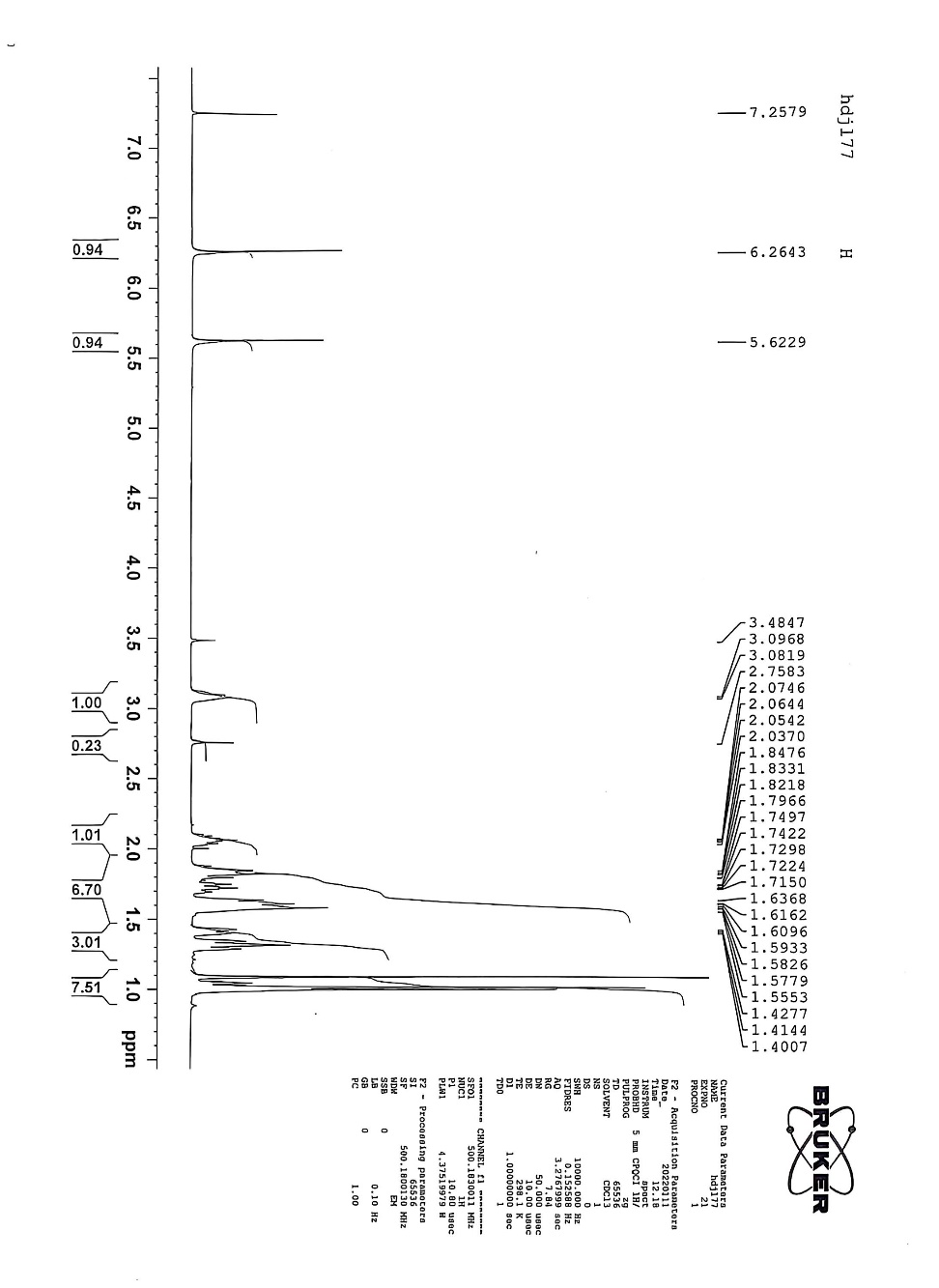


**Figure S11** ^1^H NMR spectrum of compound **6** in CDC_l3_


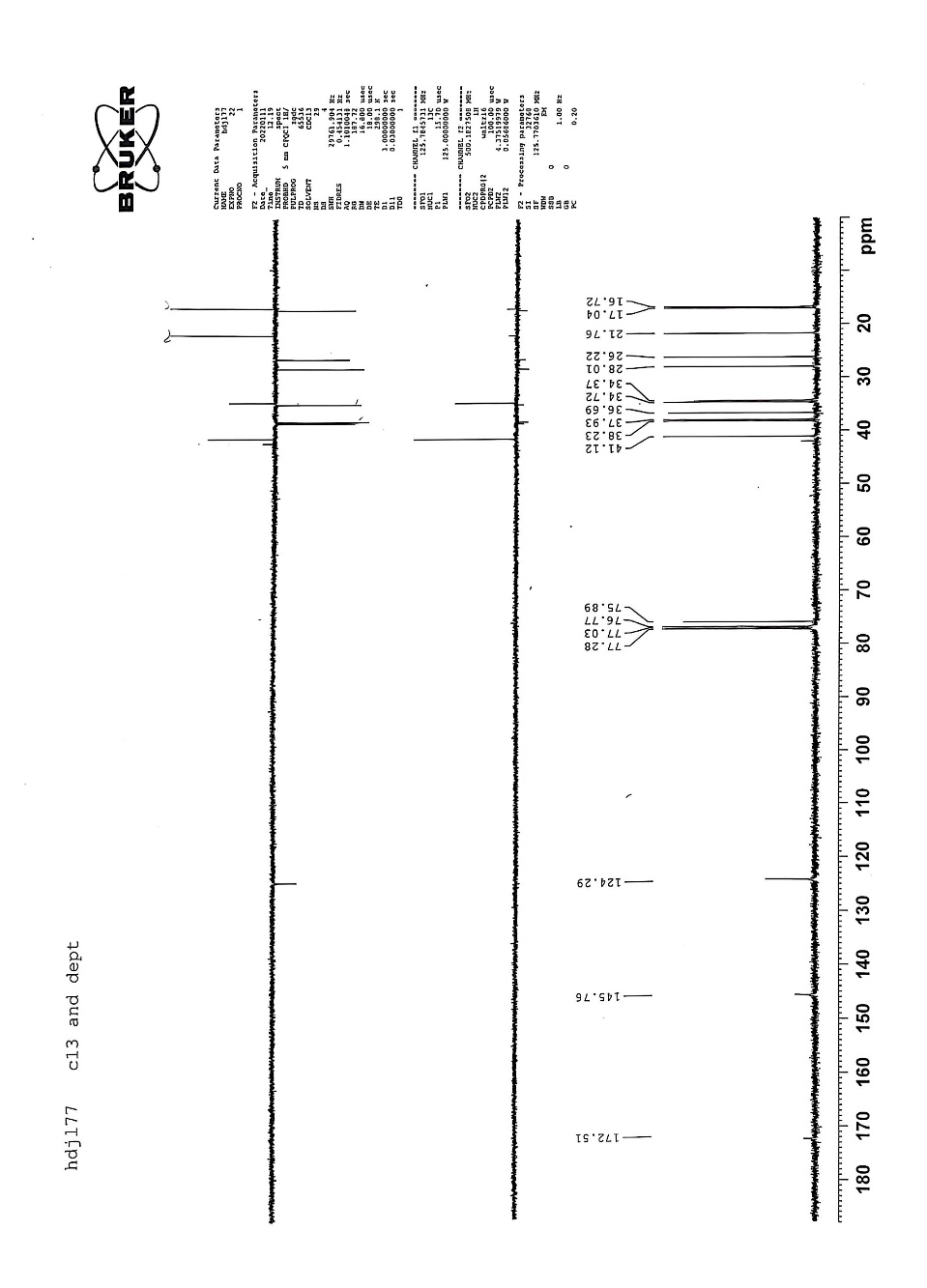


**Figure S12** ^13^C NMR spectrum of compound **6** inCDC_l3_
